# Cytosine methylation and hydroxymethylation mark DNA for elimination in *Oxytricha trifallax*

**DOI:** 10.1186/gb-2012-13-10-r99

**Published:** 2012-10-17

**Authors:** John R Bracht, David H Perlman, Laura F Landweber

**Affiliations:** 1 Ecology & Evolutionary Biology Department, Princeton University, Washington Rd., Princeton, NJ, 08544, USA; 2 Collaborative Proteomics and Mass Spectrometry Center, Molecular Biology Department and the Lewis-Sigler Institute for Integrative Genomics, Princeton University, Washington Rd., Princeton, NJ, 08544, USA

**Keywords:** epigenetics, DNA degradation, heterochromatin, methyltransferase, 5-Aza-2'-deoxycitidine, 5-azacytidine, azacitidine, decitabine

## Abstract

**Background:**

Cytosine methylation of DNA is conserved across eukaryotes and plays important functional roles regulating gene expression during differentiation and development in animals, plants and fungi. Hydroxymethylation was recently identified as another epigenetic modification marking genes important for pluripotency in embryonic stem cells.

**Results:**

Here we describe *de novo *cytosine methylation and hydroxymethylation in the ciliate *Oxytricha trifallax*. These DNA modifications occur only during nuclear development and programmed genome rearrangement. We detect methylcytosine and hydroxymethylcytosine directly by high-resolution nano-flow UPLC mass spectrometry, and indirectly by immunofluorescence, methyl-DNA immunoprecipitation and bisulfite sequencing. We describe these modifications in three classes of eliminated DNA: germline-limited transposons and satellite repeats, aberrant DNA rearrangements, and DNA from the parental genome undergoing degradation. Methylation and hydroxymethylation generally occur on the same sequence elements, modifying cytosines in all sequence contexts. We show that the DNA methyltransferase-inhibiting drugs azacitidine and decitabine induce demethylation of both somatic and germline sequence elements during genome rearrangements, with consequent elevated levels of germline-limited repetitive elements in exconjugant cells.

**Conclusions:**

These data strongly support a functional link between cytosine DNA methylation/hydroxymethylation and DNA elimination. We identify a motif strongly enriched in methylated/hydroxymethylated regions, and we propose that this motif recruits DNA modification machinery to specific chromosomes in the parental macronucleus. No recognizable methyltransferase enzyme has yet been described in *O. trifallax*, raising the possibility that it might employ a novel cytosine methylation machinery to mark DNA sequences for elimination during genome rearrangements.

## Background

DNA methylation is an epigenetic mark that alters gene expression and regulates genome stability in plants, animals and fungi [1-5]. As a stable, heritable repressive mark that is copied faithfully during DNA replication, DNA methylation of cytosine is crucial for the specification of cell lineages in mammalian and plant development [2-4,6,7] Demonstrating their essential functional roles, mutations in DNA methyltransferases (Dnmts) are lethal in mice and frogs [8-10] while mutations of the *de novo *methyltransferase Dnmt3a/b cause developmental disease in humans [11,12]. DNA methylation induces and reinforces the formation of heterochromatin, which is a tightly packed form of chromatin associated with the repression of transcription [2,4,13]. The most widely-studied regulatory methylation occurs at gene promoters, in CpG-rich regions termed 'CpG islands' [14,15], where it induces a transcriptionally silent epigenetic state that is inherited faithfully in descendant cells [4]. For example, the promoter of the stem cell pluripotency gene *Oct-4 *becomes methylated in differentiating cells, which leads to silencing and a block to reprogramming other stem cell fates in all cell progeny [16,17]. Another example is the development of the vertebrate immune system, in which cascades of transcription factors control cell fates; these fates are locked in by *de novo *DNA methylation of target gene promoters (reviewed in [18]). DNA methylation also plays a key role in oncogenic transformation: it has been known for over 15 years that cancer cell genomes display genome-wide abnormalities in DNA methylation patterns [19-23], some of which have been shown to be oncogenic [24] and to contribute to genome instability [25]. In particular, *de novo *methylation of tumor suppressor gene promoters occurs frequently in cancers, thereby silencing them and promoting transformation [19,21,22].

*Oxytricha trifallax *is a ciliated protist that performs genetic gymnastics in a complex developmental program to disentangle its genome [26]. Each cell contains two distinct types of nuclei: a germline micronucleus (MIC) that is usually transcriptionally silent, and a transcriptionally-active somatic macronucleus (MAC) that derives from the MIC but retains only 5% of its sequences [27]. The process of MAC development involves elimination of repetitive elements such as transposons [28-30] and satellite repeats [31]. In the developing, or zygotic, MAC, genes are reconstructed from relatively short segments, known as Macronuclear Destined Segments (MDSs), which are stitched together, often in a different order relative to their original order in the MIC, to produce nanochromosomes that typically contain only a single gene [26]. Genes are frequently interrupted by spacer sequences, known as Internal Eliminated Sequences (IESs), that are removed from the genome during the rearrangement process. MAC nanochromosomes are, on average, a little over 2 kb long, and there are approximately 20,000 different chromosomes in each macronucleus [26]. During the developmental process of genome rearrangement, one MIC genome irreversibly differentiates into a new, zygotic MAC, and the old, parental MAC genome degrades.

**Figure 1:**
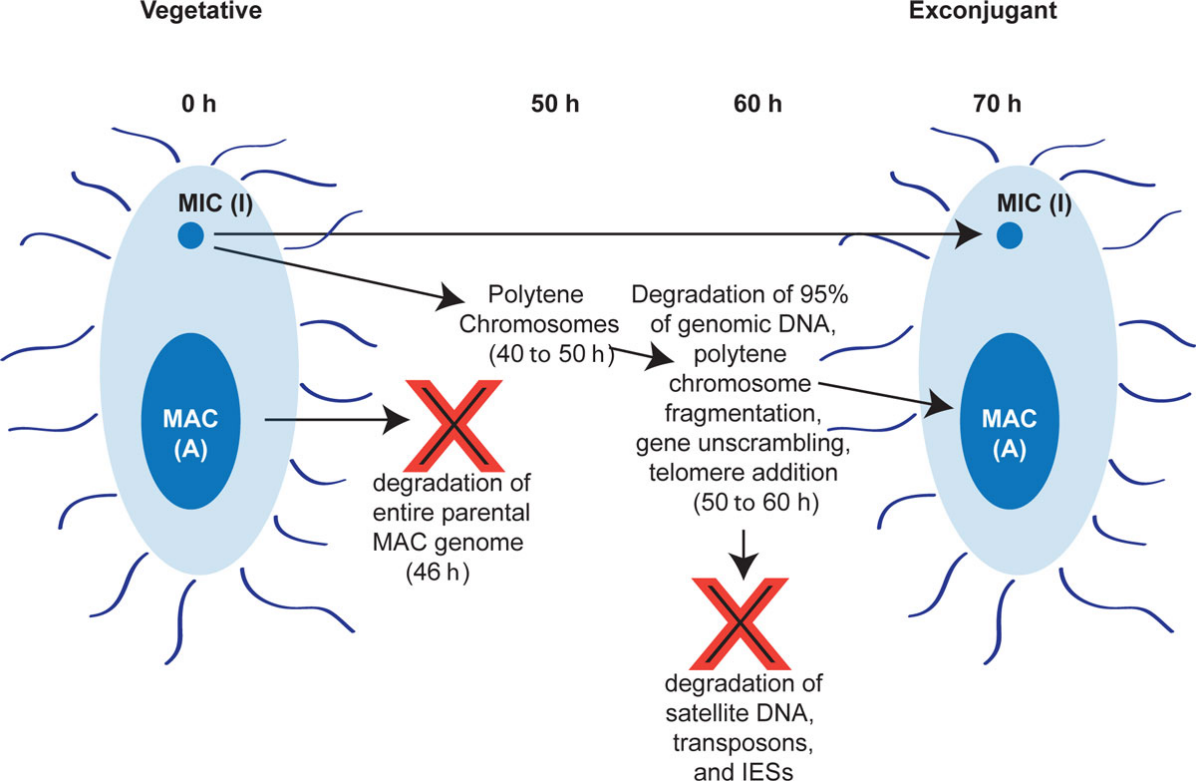
**Overview of the genome rearrangement process in *Oxytricha trifallax *and approximate timing of major events**. The two red Xs mark the degradation of two populations of DNA molecules during the genome rearrangement process. The macronucleus (MAC) and micronucleus (MIC) are both depicted.

*Oxytricha *cells thus have to eliminate two sets of DNA during development of the zygotic MAC: the entirety of the parental MAC genome and the vast majority (95%) of the MIC genome (Figure 1) [26,27,32,33]. Here we present data that implicate DNA methylation and hydroxymethylation in both DNA degradation processes. Furthermore, we describe a type of DNA methylation/hydroxymethylation whose defining characteristic is modification of every cytosine in a local region of a chromosome in a context-independent fashion, as opposed to modification of specific motifs. This pan-cytosine modification in *O. trifallax *is consistent with a model in which methylation marks DNA segments for elimination (possibly through degradation into nucleotides, which are then released into the media [34]). We present functional data linking DNA methylation to the highly regulated and essential process of genome rearrangement in *O. trifallax*. Given that no recognizable methyltransferase enzyme has yet been described in *O. trifallax*, it is possible that this unusual methylation may be deposited by novel methylation machinery.

The identification of cytosine methylation and hydroxymethylation as a degradation signal for DNA is novel although not entirely surprising, because the distantly related ciliate *Tetrahymena thermophila *uses repressive chromatin marks, including histone methylation of H3K27 [35] deposited by an Enhancer of Zeste (E(z)) homolog [36], along with several chromatin-recognizing proteins [37-39], to mark DNA for degradation. In addition, E(z) homologs (specifically, EZH2) in humans are known to recruit DNA methyltransferases [40]. However, neither DNA methylation nor hydroxymethylation has previously been implicated in a eukaryotic DNA degradation process. Therefore, the observations presented here significantly expand our understanding of functional roles for DNA methylation and hydroxymethylation in biology.

## Results

### Immunofluorescence reveals that cytosine methylation is specific to conjugation

**Figure 2:**
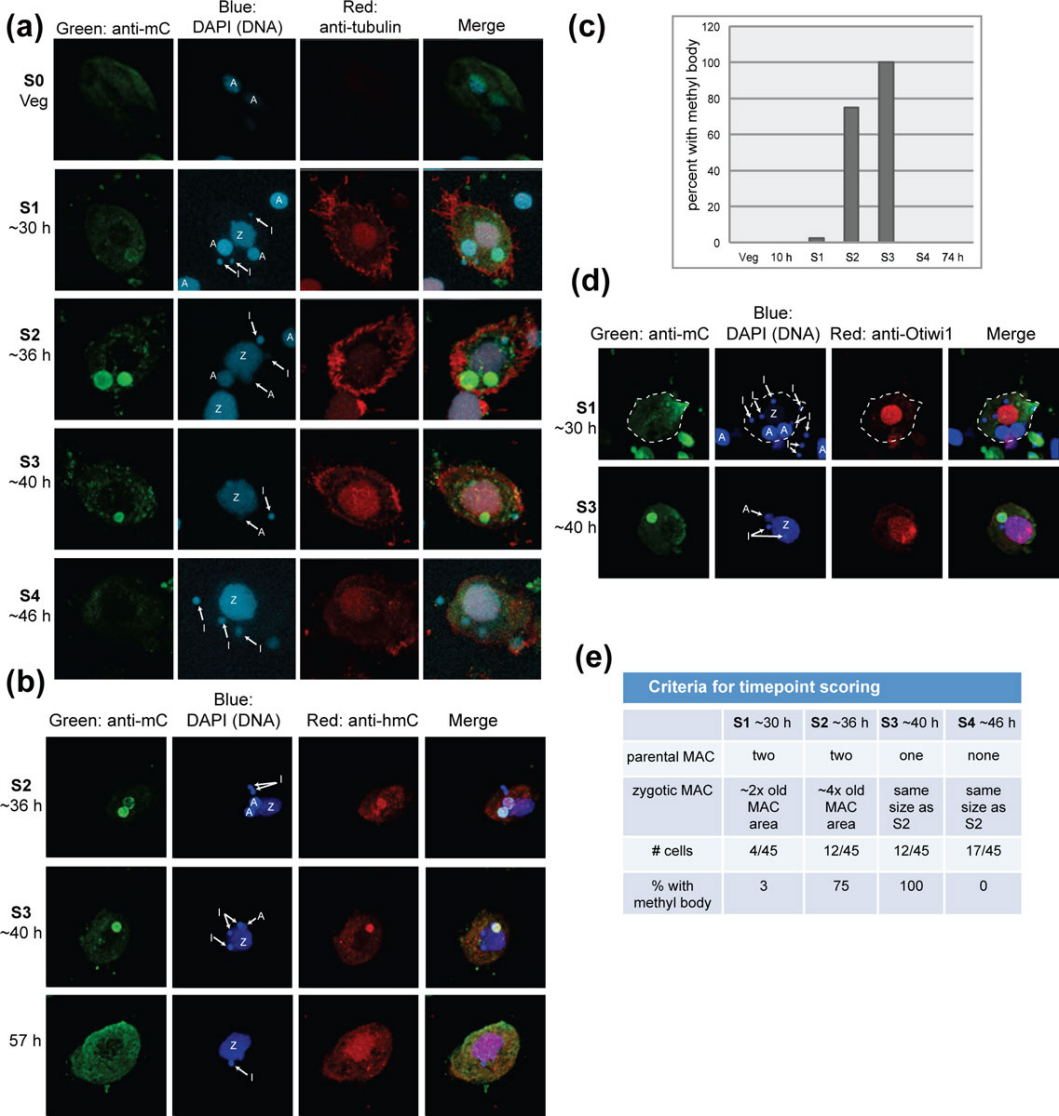
**Immunofluorescence of fixed *Oxytricha trifallax *cells during genome rearrangements**. **(a) **A methylcytosine signal appears during the 30-40 h (hour) window. Cells are staged by the nuclear morphology observed in single, well-timed cells. The micronucleus (I), parental macronucleus (A) and zygotic macronucleus (Z) are all indicated. **(b) **Co-immunofluorescence analysis shows co-localization of methylcytosine and hydroxymethylcytosine. **(c) **Quantification of methylcytosine-containing DNA-rich bodies (the parental macronucleus) in the 46 h population shown in (a), separated by cell stage. **(d)**. Co-immunofluorescence with methylcytosine and anti-Otiwi1, a temporal marker for zygotic macronucleus development. Note the temporal separation of Otiwi1 and DNA methylation during the transition between S1 and S3. **(e) **The nuclear morphology criteria used in (a) - (d) for staging cells. MAC, macronucleus; Veg, vegetative.

To investigate the role of cytosine methylation in genome rearrangements, we harvested cells 46 h (hours) post-conjugation, fixed them and performed immunofluorescence against 5-methylcytosine (Figure 2a). Methylcytosine immunofluorescence signal was observed only in the degrading parental macronucleus of 46 h exconjugant cells and not in vegetative cells (Figure 2a). In the 46 h population of cells, only half contained detectable methyl-cytosine signal above background. However, it was possible to sort these cells by developmental stage based on nuclear morphology, and we designate these internal stages S0, for vegetative cells, and S1 through S4 for conjugating cells (see Figure 2e for criteria). Because *O. trifallax *cultures cannot be perfectly synchronized, the oldest cells (S4) are 46 h post-conjugation, but other cells are younger, and the youngest cells, a minority of the population, are approximately 30 h post-conjugation (S1), as determined by co-immunofluorescence with the temporal marker Otiwi1, a PIWI homolog (Figure 2d). These data show that methylcytosine does not localize in parental MAC until after Otiwi1 immunostaining is strongly reduced at approximately 40 h. In between S1 and S4 it is possible to distinguish two more stages based on the number of parental macronuclei and the size of the developing zygotic macronucleus (Figure 2a,b,e). These stages (S2 and S3) appear to correspond to approximately 36 and 40 h post-conjugation, respectively. Notably, the percentage of cells exhibiting cytosine methylation of the parental macronucleus rises to 75% and 100% in S2 and S3 cells, respectively, but drops to zero when the parental macronucleus is completely eliminated in S4 (46 h) cells (Figure 2a,b,e). These data suggest a model in which DNA degradation is signaled by significant cytosine methylation. Modified chromosomes of the parental macronucleus are eliminated along with their epigenetic DNA modifications during the degradation process. Due to the imprecision of the synchrony of *O. trifallax *cells, this process can be captured in the range of cells observed at a single timepoint (46 h) post-conjugation; the full methylation-degradation process appears to occupy only approximately 8-10 h (Figure 2e).

Hydroxymethylcytosine is an epigenetic mark only recently recognized as a biologically important modification, with roles distinct from DNA methylation [41,42]. We performed immunofluorescence with an anti-hydroxymethylcytosine antibody in *O. trifallax *(Figure 2b) and found detectable levels of this modification that overlap completely with DNA methylation (Figure 2b). In general, the methylcytosine immunofluorescense signal is more robust, but hydroxymethylcytosine consistently localizes to the same parental MAC in cells approximately 36-40 h post-conjugation. While methylation was not observed in any cells after S4 (when the parental MAC is eliminated), we consistently noted a faint hydroxymethylation signal in the zygotic MAC at this late stage cell (see the 57 h cell in Figure 2b).

### Detection of cytosine methylation and hydroxymethylation by mass spectrometry

**Figure 3:**
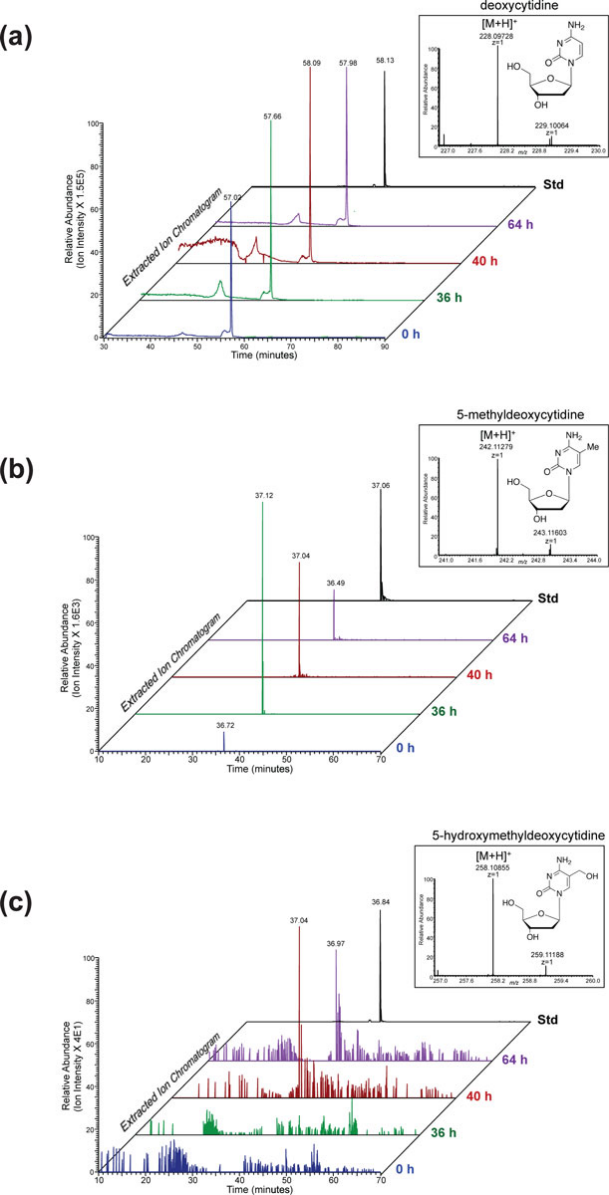
**Direct detection of 5-methylcytidine and 5-hydroxymethylcytidine in *Oxytricha trifallax *DNA using high-resolution nano-flow UPLC-mass spectrometry**. Nucleosides generated from purified *O. trifallax *DNA isolated 0 h, 36 h, 40 h and 64 h post-conjugation, or from standards (synthetic PCR products containing either unmodified nucleosides, 5-methylcytidine or 5-hydroxymethylcytidine), were subjected to LC-MS on a high-resolution nano-flow UPLC - Orbitrap mass spectrometry platform. Extracted chromatograms of the **(a) **cytidine, **(b) **5-methylcytidine and **(c) **5-hydroxymethylcytidine [M+H]^+ ^ions are shown, displaying a single prominent peak for each molecular species across the chromatographic timescale. Inset into the chromatograms are mass spectra of the detected [M+H]^+ ^ion for each molecular species; empirical mass measurements for these ions were each within ±0.0005 Da of theoretical values for cytidine, 5-methylcytidine and 5-hydroxymethylcytidine atomic compositions.

The detection of cytosine methylation in organisms lacking methyltransferase enzymes has proven contentious. Because the modifications reported here are transient, and because we have not yet identified a cytosine methyltransferase enzyme in *O. trifallax*, we definitively confirmed the presence of cytosine methylation and hydroxylmethylation through direct detection by ultra high performance liquid chromatography (UPLC)-high-resolution mass spectrometry (MS) (Figure 3). To accomplish this, we subjected genomic DNA harvested from *O. trifallax *at various time points post-conjugation to degradation into nucleosides by treatment with nuclease and phosphatase enzymes. As a positive control and standard for the detection of 5-methylcytidine, 5-hydroxymethylcytidine and cytidine, we used enzymatically degraded PCR products obtained from PCR reactions containing fully cytosine-methylated, hydroxymethylated or non-methylated constituents. We subjected these free nucleosides to UPLC-MS using a capillary nano-flow UPLC system hyphenated to a high mass accuracy, high-resolution Orbitrap-based MS platform. This approach yielded chromatographic resolution of the nucleosides and modified nucleosides into their characteristic reversed-phase elution profiles, and enabled unambiguous confirmation of nucleoside identities by the accurate mass measurement of the Orbitrap mass spectrometer, which specified their atomic composition.

*O. trifallax *samples not only displayed the presence of both 5-methylcytidine (Figure 3b) and 5-hydroxyl-methylcytidine (Figure 3c), but also revealed temporal dynamics in abundance that were similar to those observed by immunofluorescence (Figure 2a,b). Relative 5-methylcytidine amounts increased sharply 36 h post-conjugation over vegetative levels, and then underwent a progressive decrease at the 40 h and 64 h time points (Figure 3b). In contrast, 5-hydroxylmethylcytidine was undetectable at the 36 h time point and only became detectable at 40 h post-conjugation, remaining elevated through the 64 h time point (Figure 3c). Both differed from unmodified cytidine levels, which were expectedly high and approximatelu equivalent throughout all time points (Figure 3a). The mass spectrometry data are publicly available at OxyDB, the *O. fallax *genome database [43].

### Deep sequencing of methylated DNA in the macronucleus and micronucleus

The immunofluorescence results and confirmation by mass spectrometry motivated a genome-wide search for sequences that become methylated specifically during genome rearrangement. We chose to use methyl-DNA immunoprecipitation coupled with deep sequencing (meDIP-seq) [44-52] to identify specific locations in the genome enriched for methylcytosine or hydroxymethylcytosine. Two Illumina sequencing libraries were constructed, one from vegetative cells, to act as a non-methylated/non-hydroxymethylated control, and one from 46 h post-conjugation DNA isolated from the same cells imaged in Figure 2a. Immunoprecipitation was performed as described [44], with either an IgG control, the methylcytosine antibody used for immunofluorescence (Figure 2) [53] or an antibody to hydroxymethylcytosine [54]. The immunoprecipitated material was subjected to high-throughput sequencing, from which between 5 million and 9 million reads were obtained from each library, sufficient to provide 10- to 20-fold coverage of the MAC genome. The mapped reads were normalized for total read number (sequencing depth of each library) and chromosome/contig length, providing a reasonable abundance estimate for each genomic sequence (as reads per kb per million reads, or RPKM).

**Figure 4:**
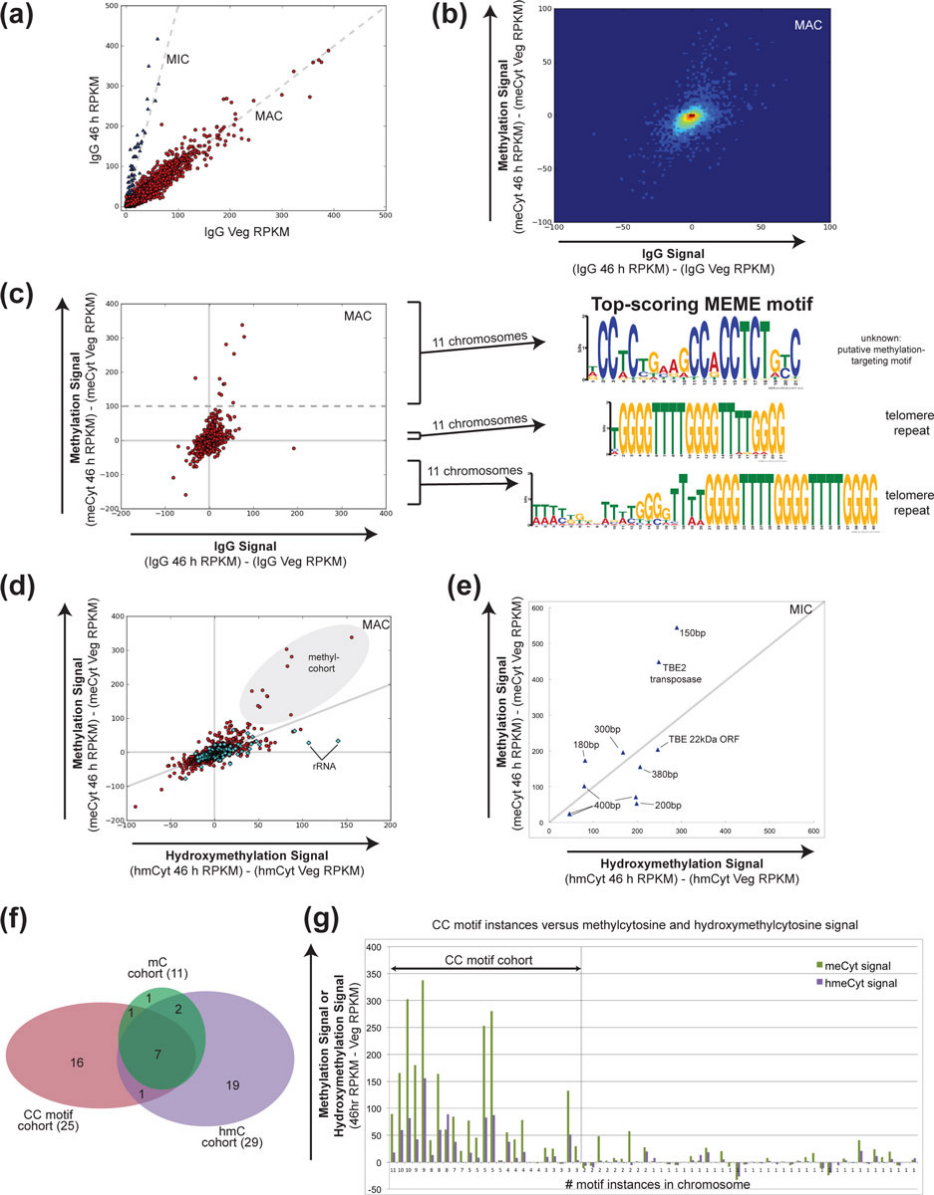
**Methyl-DNA-immunoprecipitation sequencing (meDIP-seq) analysis of DNA from 46 h conjugating cells shown in Figure 2**. All reads are presented in reads per kb per million (RPKM) to correct for length of sequences and sequencing depth. **(a) **IgG controls for both vegetative (x-axis) and 46 h DNA (y-axis). Micronuclear sequences are polyploid at the 46 h stage due to polytenization of chromosomes prior to genome rearrangements, and fall along a 5:1 gradient, shown as a dashed line. **(b) **Heatmap of methylcytosine immunoprecipitation (meCyt) reads with vegetative reads subtracted; IgG on the x-axis and methylcytosine on the y-axis. **(c) **A scatter plot to highlight the outliers along the y-axis in (b). The dotted line denotes the threshold (100 excess reads in 46 h meCyt) used to define the methylation cohort. These 11 chromosomes were fed into the MEME algorithm, which identified the CC motif on the right, which was highly statistically significant (MEME e-value = 2.8e-236); control cohorts of chromosomes were selected from the un-enriched population (middle) and depleted population (bottom): no motifs were found and the highest scoring motif in these cases was the telomeric sequence, G_4_T_4_G_4_T_4_G_4_. **(d) **Hydroxymethylcytosine (x-axis) versus methylcytosine (y-axis) immunoprecipitation data. Nanochromosomes encoding ribosomal proteins or the ribosomal RNA are shown as cyan diamonds; the rest of the nanochromosomes in the genome are plotted as red circles. Note co-enrichment of the methylation cohort with both methyl- and hydroxymethyl- modifications, and that the chromosomes encoding ribosomal RNA and ribosomal proteins are only enriched for hydroxymethylcytosine. **(e) **The same analysis as (b) and (c), but for micronuclear contigs separated from the genome assembly in (a). Representative satellite repeats (labeled with their repeat unit length) and TBE elements display a complex mixture, suggesting a heterogeneous combination of DNA modifications in the genome. The strong hydroxymethylcytosine signal for the 170 bp satellite repeat (10,953 hmCyt reads, x-axis; 4,166 meCyt reads, y-axis; Additional file 3) placed it outside the bounds of this figure. **(f) **Venn diagram of the methylation (mC) cohort, hydroxymethylation (hmC) cohort and CC motif cohort. **(g) **All 69 CC motif-containing chromosomes plotted with their methylation and hydroxymethylation signals from meDIP-seq data. IgG, immunoglobulin G; MEME, Multiple Em for Motif Election; TBE, telomere bearing element; Veg, vegetative.

To establish the accuracy of our approach we plotted RPKM from vegetative IgG versus 46 h IgG, thereby visualizing the difference in copy number between conjugating and vegetative cells (Figure 4a). In this chart, each chromosome is represented by a point on a scatterplot, and two patterns are evident: a 46 hr-to-vegetative line with a slope of 1 (*R*^2 ^= 0.937), which contains the MAC chromosomes of *O. trifallax *at equal abundance in both vegetative and 46 h cells, and other sequences that are approximately five-fold enriched at 46 h relative to vegetative DNA (*R*^2 ^= 0.965) (Figure 4a). The 46 hr enriched sequences (blue triangles in Figure 4a) comprise MIC sequences that have not been filtered out of the MAC genome assembly; they lack terminal telomeres and consist of either repetitive satellite sequences or TBE (telomere bearing element) transposons, a *Tc1/mariner *class of transposons abundant in the *O. trifallax *micronuclear genome and eliminated during genome rearrangements.

During the *O. trifallax *sexual cycle (Figure 1), the MIC genome becomes amplified into polytene chromosomes prior to genome rearrangement and fragmentation into nanochromosomes. Published work supports polytenization of approximately 15-fold, reaching maximum amplification 40-50 h post-conjugation [26,33,34,55,56]. Given that our data suggest that the amplification at 46 h post-conjugation is approximately five-fold higher than that of vegetative cells, we conclude that our conjugating *O. trifallax *population had not reached full polytenization at the 46 hr timepoint, but that it was within two DNA replication cycles of maximal amplification. In total, there were 58 MIC-limited 46 hr-enriched contigs in the genome assembly, and these were extracted from the MAC genome and analyzed separately in all sub-sequent analysis. The ability to separate out known (and novel) MIC contigs from the MAC genome assembly confirms the general accuracy of the meDIP-seq approach for measuring DNA levels in a sample. Therefore, we turned next to analysis of the methylcytosine and hydroxymethyl-cytosine immunoprecipitation data.

**Figure 5:**
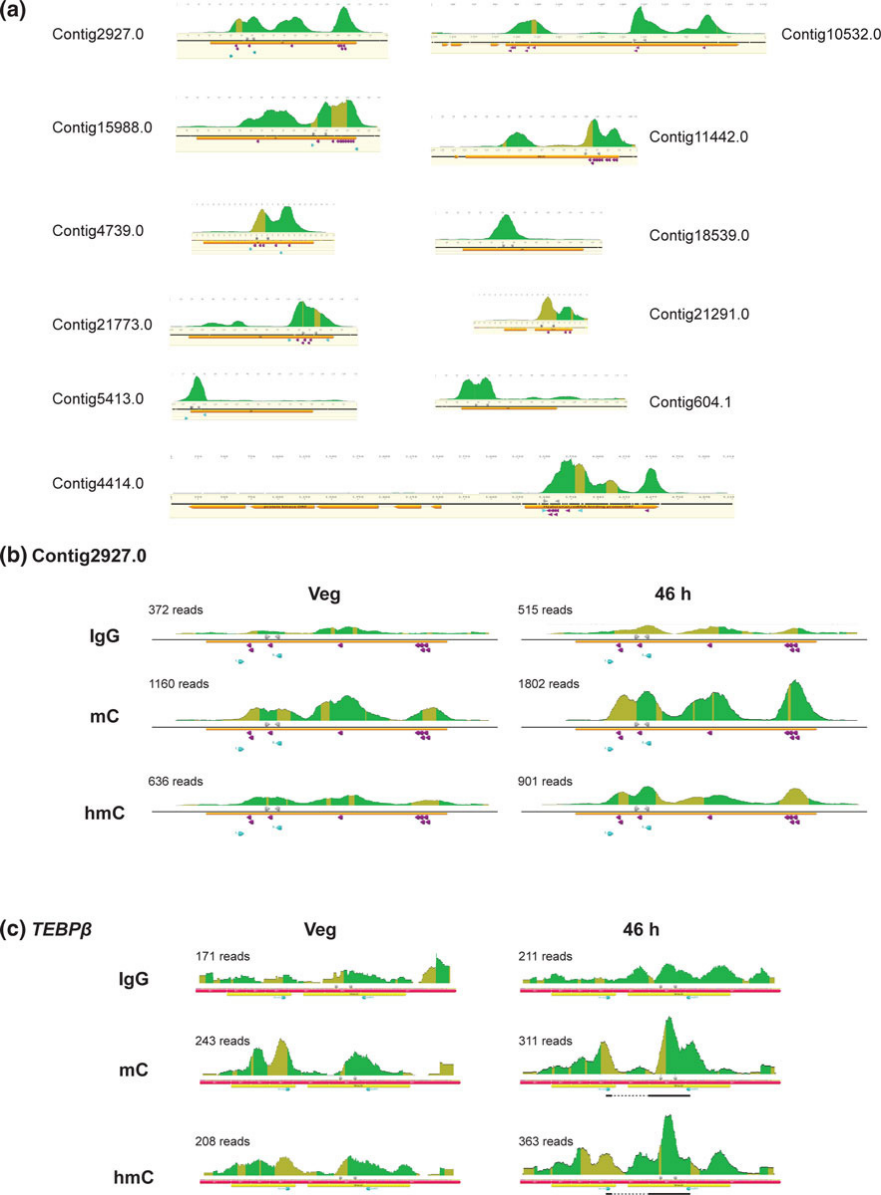
**Visualization of meDIP-seq data mapped to select *Oxytricha trifallax *chromosomes**. **(a) **46 hour methylcytosine immunoprecipitation reads mapped to the methylation cohort of eleven chromosomes (Figure 4c, above the dotted line). Read depth is represented by the peaks in the y dimension for each chromosome (scale not comparable between chromosomes). CC motifs are shown as purple arrowheads below the reads for each chromosome. The gold bar represents the ORF, consistently oriented from left to right in all chromosomes. Teal arrows indicate oligos used in bisulfite-PCR, while grey arrows shown above the ORF indicate oligos used in bisulfite-qPCR. **(b) **Scaled plot of Contig2927.0 meDIP-seq signal, the highest ranking chromosome in both the methylation and hydroxymethylation cohorts, for immunoprecipitation with IgG, methylcytosine (mC) and hydroxymethylcytosine (hmC) in both vegetative (negative control) and 46 h DNA. One million reads from each library were plotted at equal scales, so that the heights of peaks (and read numbers) are directly comparable. **(c) **Scaled plot of *TEBP**β ***showing enrichment both for methylcytosine (mC) and hydroxymethylcytosine (hmC), with plotting and scaling as in (b). Dark lines under 46 h mC and hmC plots represent the methylated/hydroxymethylated aberrantly spliced product identified by bisulfite-PCR (shown in Figure 6d,e). ORF, open reading frame; qPCR, quantitative PCR; *TEBP**β***, *Telomere End-Binding Protein ****β***; Veg, vegetative.

Analysis of the meDIP data is complicated by two factors: firstly, the tendency of the methylcytosine and hydroxy-methylcytosine antibodies to bind, albeit weakly, to unmodified cytosine; and, secondly, the tendency of a given genomic sequence to bind non-specifically to the beads or to constant portions of the antibody. These phenomena generate noise in the meDIP-seq data (Figure 5b,c), which was filtered out in a two-step normalization process by taking advantage of the fact that neither methylcytosine nor hydroxymethylcytosine is detected in vegetative cells (Figures 2a and 3b,c). In the first step, the 46 hr reads were normalized for total read number and chromosome length, to give an RPKM value. In the second step, the RPKM value obtained with the same antibody from the vegetative cells, in which methylation and hydroxymethylation were not present, was subtracted from the first value. This normalization procedure was performed for the methylcytosine, hydroxymethylcytosine and IgG data, removing the majority of the noise in all three datasets. We designate each residual dataset, obtained by subtracting vegetative reads from 46 h reads, the 'signal' for the corresponding antibody (methylcytosine, hydroxymethylcytosine or IgG).

In examining the methylcytosine versus IgG signal in MAC, most chromosomes are clustered at zero on both axes (Figure 4b). Surprisingly, these data suggest that the majority of the MAC genome does not display detectable levels of cytosine methylation at 46 h. However, a skew of MAC chromosomes exhibiting an excess of reads from the 46 h sample is evident (Figure 4c). There is a natural break in the distribution separating eleven chromosomes with over 100 excess reads (dotted line in Figure 4c) in the methylcytosine library. We therefore separated these chromosomes into a methylation cohort group for further analysis. This group of chromosomes encodes several predicted proteins of potential functional interest (Additional file 1), including DNA-binding proteins (an Alba protein, a zinc finger protein, and a TFIIA transcription factor), RNA-binding proteins (an RRM (RNA recognition motif)-containing protein and an LSm (Sm-like) domain-containing protein), and protein kinases. However, the reasons why these chromosomes might be preferentially methylated were not immediately clear from the intial analysis of our meDIP data. We therefore further analyzed these methylcytosine-enriched chromosomes for specific motifs by using the Multiple Em for Motif Election (MEME) software package [57].

A highly significant 20 bp-long pyrimidine-rich motif (MEME e-value = 2.8e-236) was identified in the methylation cohort chromosomes and not detected in control cohorts of eleven MAC chromosomes lacking enrichment in 46 h DNA (selected from the center of the distribution, the middle group in Figure 4c) or enriched in vegetative DNA (bottom group in Figure 4c). Due to the recurrence of CC in this motif, we termed it the 'CC motif'. The motif appears to be bipartite, with strong C-rich signals reaching maximum information content approximately 10 bp apart, which suggests a separation of one turn of the DNA helix (Figure 4c). The CC motif was present an average of five times on each methylation cohort chromosome, generally occurring in clusters (59 statistically significant motifs were present on eight of the eleven chromosomes). Of additional interest was the apparent correlation between the CC motif and the meDIP-seq data for those eight chromosomes that contained the motif; the motif mapped, in most cases, to the majority of observed peaks (Figure 5a). Three of the eleven methylation cohort chromosomes did not contain the motif, and it may be that these chromosomes instead contained more divergent instances of the motif that were less confidently identified by MEME, although the existence of other methylation-targeting motifs cannot be excluded. The fact that some meDIP-seq peaks did not correlate with the motifs in Figure 5a also supports the idea that additional methylation-targeting motifs remain unidentified in the analysis presented here.

To investigate the potential role of the CC motif further, we carried out a genome-wide scan to identify additional occurrences, using a stringent false-discovery rate of 1 × 10^-7 ^(that is, one false discovery per 10 million occurrences of the motif). Genome-wide, we observed 229 instances of the motif, on a total of 69 chromosomes, 61 of which were not in the methylation cohort. Furthermore, we observed that chromosomes with 3 or more CC motifs were also enriched for both methylcytosine and hydroxymethylcytosine at the 46 h timepoint (Figure 4g), so we label these 25 chromosomes (with 3 or more motifs) the CC motif cohort (Additional file 2).

The CC-motif cohort includes additional, potentially informative candidates, including 3 new *Alba *genes, bringing the total identified in our methylation analysis to five (two were in the methylation cohort, Additional file 1). There was an overall enrichment in DNA- and RNA-binding proteins in this CC motif cohort (Additional file 2). Strikingly, both *O. trifallax *genes encoding fibrillarin were identified in the CC-motif scan, with 3 CC motifs each (Additional file 2).

The hydroxymethylcytosine immunoprecipitation data yielded a similar picture to the methylcytosine immuno-precipitation, but there were also important differences. We performed a ranking of MAC chromosomes by their hydroxymethylcytosine signal, with minimum thresholds of 40 excess 46 h reads and a hydroxymethylation-to-IgG signal ratio of 1.5 (Additional file 3). Remarkably, we found that 9 of 11 methylation cohort chromosomes were also present in the hydroxymethylation cohort of 29 MAC genes (Additional file 3). The top member is the same on both lists (unknown protein-encoding chromosome Con-tig2927.0), but the other overlapping members are mostly present in a slightly different order. In addition, several new chromosomes appear in the hydroxymethylation cohort, with ribosomal RNA chromosomes (two isoforms of the same genetic sequence) appearing second and third. Three ribosomal protein-encoding genes also appeared in the hydroxymethylation but not the methylation cohort. Several additional ribosomal protein genes were relatively more hydroxymethylated than methylated (Figure 4d, cyan diamonds).

The MIC genome contains many short repeats (J.R.B., L.F.L, manuscript in preparation). One of the most abundant has a 170 bp repeat unit that is completely eliminated from the developing macronucleus during genome rearrangement [31]. This repeat was strongly enriched in hydroxymethylcytosine meDIP data (Additional file 3). In addition, several other satellite repeats were also significantly enriched for hydroxymethylcytosine relative to methylcytosine (Additional file 3). Methylation was detected strongly on a different set of MIC sequences comprising different satellite repeats (shown in Additional file 1 and Figure 4e, with their repeat unit lengths) and a TBE2 transposase sequence (Additional file 1, Figure 4e). We conclude that repetitive MIC-limited sequences, such as satellite DNA and transposons, may be preferentially modified by hydroxymethylcytosine or methylcytosine, or both (Figure 4e, Additional files 1 and 3).

The meDIP sequencing read data and genome-wide mapping analysis are publicly available [GEO: GSE41060].

### Bisulfite PCR confirmation of meDIP-seq results

In order to validate the meDIP-seq results, we turned to the gold standard bisulfite-sequencing technique to examine the methylation patterns at single-base pair resolution for a few predicted chomosomal loci. Bisulfite treatment of DNA induces deamination of cytosine to uracil, which is sequenced as thymine [58]. The deamination of cytosine to uracil is blocked by methylation [58,59]; therefore, when analyzing PCR product sequences from bisulfite-treated samples, we infer that any cytosines that are not changed to thymine must have been methylated originally. It is important to keep in mind that methylcytosine and hydroxymethylcytosine are indistinguishable by bisulfite sequencing [60]. We performed bisulfite-PCR on two independent *O. trifallax *samples: the 46 h DNA used for meDIP and an independently staged 40 h DNA sample. As a negative control, we used vegetative *O. trifallax *DNA from strains JRB310 and JRB510, which are compatible mating types that are mixed to initiate conjugation. In most organisms, cytosine methylation occurs at specific motifs (CpG, CpHpG or CpHpH), so standard bisulfite-sequencing oligos are designed with non-CpG cytosines converted to thymine. However, we observed virtually no methylation (less than 0.5% of cytosines) in bisulfite-converted 40 h DNA with the cytosine-to-thymine converted oligos (Additional file 4a). However, using cytosine-retaining oligos for PCR of bisulfite-converted 40 h or 46 h DNA yielded detectable bands that were not observed in vegetative DNA; sequencing of these bands confirmed heavy non-CpG methylation (Additional file 4b). The methylation levels were variable but quite high: cytosine residues in Contig4414 were 91% methylated on the forward strand and 84% on the reverse, suggesting a potential strand bias for modification.

**Figure 6:**
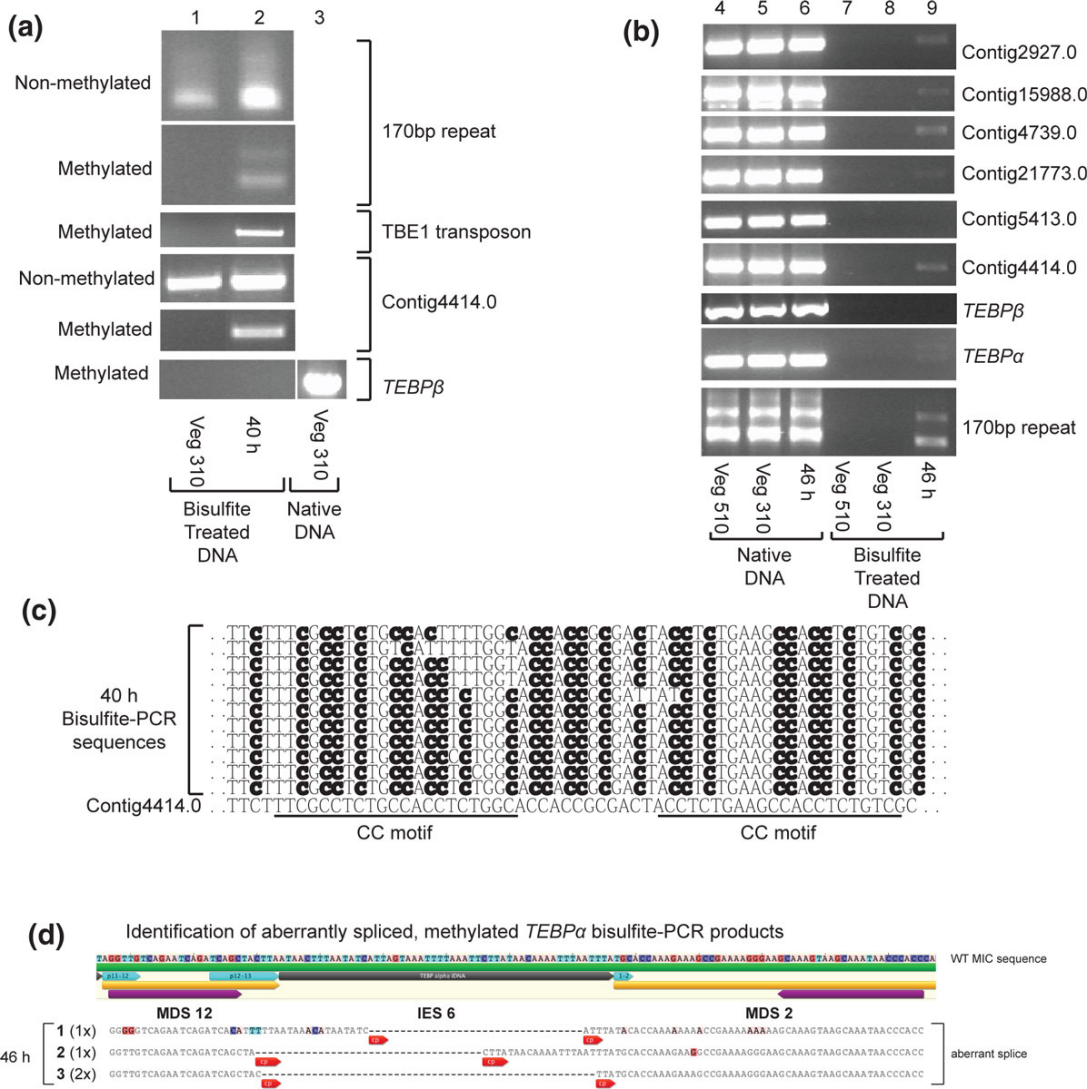
**Confirmation of predicted methylation by bisulfite sequencing**. **(a) **Use of C-to-T converted primers specifically amplifies the non-methylated chromosomes from bisulfite-treated 40 h (hour) or vegetative DNA (labeled 'Non-methylated'), while standard, cytosine-retaining primers amplify methylated DNA (labeled 'Methylated'). PCR of *TEBP**β ***was performed on native DNA to demonstrate functionality of the oligos. **(b) **A repeat of the experiment in (a), but with a 46 h, not 40 h, sample, and with additional methylation cohort chromosomes, as well as *TEBP**α ***and *TEBP**β***. Strains JRB310 (310) and JRB510 (510) are two mating types of *Oxytricha trifallax *whose mixing induces conjugation; the 40 h and 46 h samples are an equal combination of both mating types. **(c) **Bisulfite sequencing of eleven Contig4414.0 clones. Cytosines in bold are methylated. Note that methylation occurs in all sequence contexts and can have runs of consecutive skipped residues. Two CC motifs occur in this region of the chromosome, as marked. **(d) **Three aberrantly spliced, and methylated/hydroxymethylated, versions of *TEBP**α ***identified by bisulfite-PCR of 46 h DNA. MDS 12 would normally never be fused directly to MDS 2, as observed in these products; 3 to 4 bp cryptic pointers (marked 'cp' in red arrowheads) are present at recombination junctions. Normal unscrambling entails fusion of MDS 1 to MDS 2 and MDS 12 to MDS 13; wild-type pointers for these events are marked in turquoise arrowheads. Products 2 and 3 (recovered 1 and 2 times, respectively) appear heavily methylated, while product 1 is more lightly methylated (G to A substitutions indicate C to T conversions on the opposite strand, highlighted in pink). Colored nucleotides differ from the WT sequence (top). PCR primers are marked by purple arrowheads. MDS, Macronuclear Destined Sequence; *TEBP**α***, *Telomere End-Binding Protein ****α***; Veg, vegetative.

**Figure 7:**
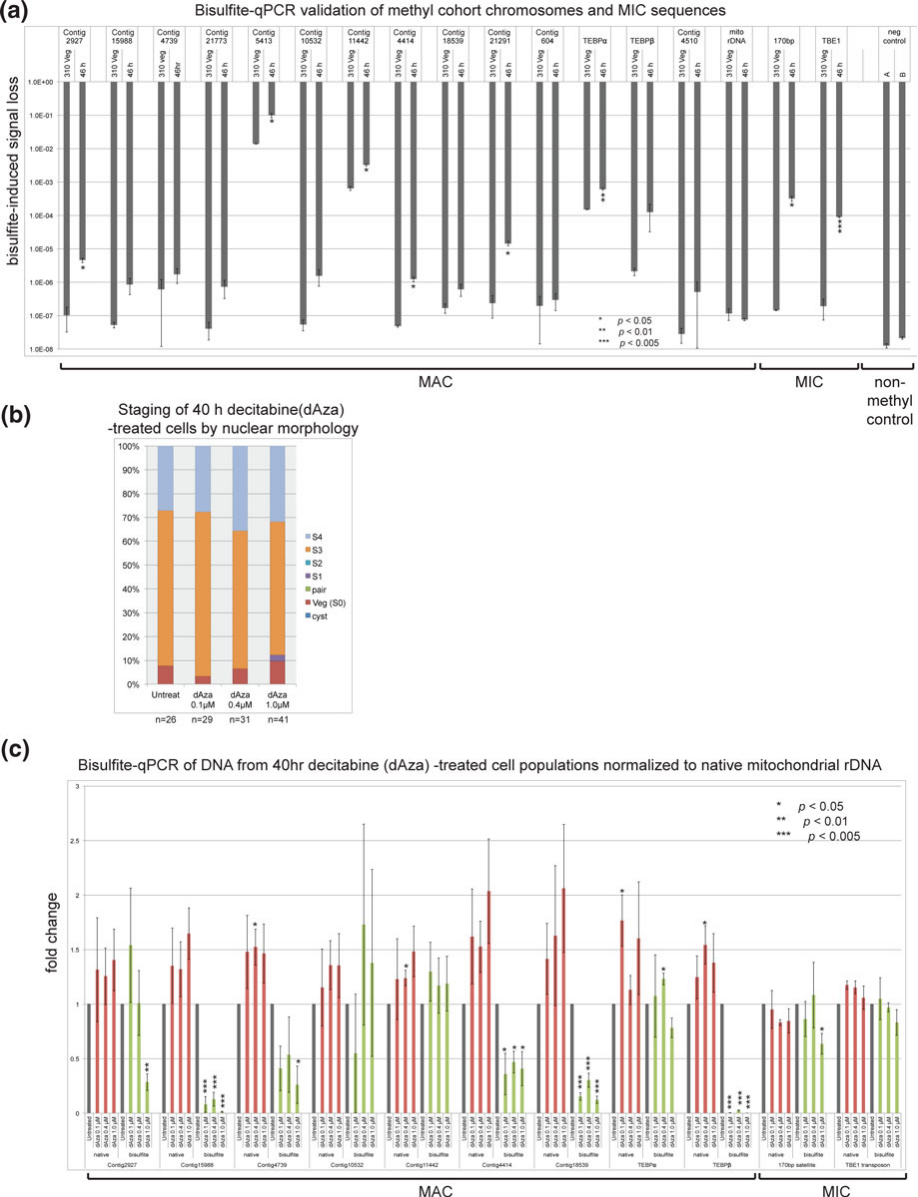
**The use of bisulfite-qPCR to detect methylated/hydroxymethylated DNA and loss of methylation after decitabine treatment**. (a) Validation of bisulfite-qPCR for *Oxytricha trifallax *DNA. The ddCt method was used to quantify the loss of signal induced by bisulfite treatment relative to signal from an equal amount of untreated DNA. Signal was normalized to total DNA used as input for the bisulfite treatment. A PCR fragment of Contig4414 amplified from vegetative (nonmethylated) DNA provided the conversion control (negative control for methylation); two indepdendent bisulfite treatments of this PCR product, A & B, were used as templates in qPCR. All qPCR was performed in triplicate and the average is plotted with standard error. The Student's 1-tailed *t*-test for unequal variance was used and *p*-values are indicated: *, *p *< 0.05; **, *p *< 0.01, ***, *p *< 0.005. Values marked *** in Figures 7 and 8 appear significant even with a correction for multiple tests. **(b) **Staging data for 40 h decitabine (dAza)-treated cells. Cells were fixed and DAPI stained to allow staging based on nuclear morphology as in Figure 2e. **(c) **Bisulfite-qPCR analysis of decitabine-induced demethylation in 40 h cells. Both native (red bars) and bisulfite-converted DNA (green bars) are shown, normalized to native mitochondrial rDNA signal (for loading) and to untreated cells (grey bars) to determine fold change. All qPCR was performed in triplicate and the average is plotted with standard error. Statistical test for significance was carried out with Student's 1-tailed *t*-test (*, *p *< 0.05; **, *p *< 0.01, ***, *p *< 0.005). DAPI, 4',6-diamidino-2-phenylindole; qPCR, quantitative PCR; Veg, vegetative.

Given the meDIP-seq data prediction of hydroxymethylation of the 170 bp satellite repeat (Additional file 3), we also tested the 170 bp repeat by bisulfite-PCR and confirmed that this repeat is highly modified in 40 h or 46 h cells, but no band is detectable in bisulfite-treated DNA from vegetative cells (Figures 6a,b and 7a). Sequencing of these PCR products showed that the 170 bp satellite was methylated or hydroxymethylated at 71% and 54% of forward- and reverse-strand cytosines, respectively (Additional file 4c). The same pattern held for the well-known and abundant TBE1 family of transposable elements (Figure 6a), confirmed by clone sequencing (Additional file 4e). While the TBE1 transposon sequences were not sufficiently enriched in meDIP-seq reads to be included in Additional file 1, either the transposase or 22 kDa ORF [61] encoded in the related TBE2 transposon were present in the methylation or hydroxymethylation cohorts, respectively. One explanation for the absence of TBE1 from these cohorts may be the lower sensitivity of meDIP-seq relative to bisulfite-PCR sequencing.

When we analyzed the sequencing traces from bisulfite-PCR of 40 or 46 h cells, the methylation (or hydroxymethylation) was present at all residues within the amplified regions of most molecules, interspersed with occasional, apparently unmodified cytosine residues (Figure 6c). Careful examination of patterns of modification in Contig4414.0 (Figure 6c), or the MIC-limited 170 bp satellite repeat (Additional file 4c) or a TBE1 transposon (Additional file 4e), revealed runs of 3 to 8 consecutive unmethylated cytosines in some sequencing reads. This tendency of the putative methyltransferase to skip some consecutive cytosines suggests either that the activity is processive and may occasionally fall off the DNA, or that methylation could potentially be blocked by an unknown DNA-binding factor or different modification at these sites, effectively leaving a footprint. However, since every cytosine displayed either methylation or hydroxymethylation in at least some DNA molecules, we infer that any block to methylation is not consistent across the chromosomes. The observation that methylation occurred regionally, with high consistency on cytosines in all sequence contexts, suggests that deposition may occur in a processive fashion. These observations are also consistent with reports of high-density methylation in all sequence contexts of local genomic regions in the fungi *Neurospora crassa *[62] and *Uncinocarpus reesii *[1].

The identification of an enriched motif in our data, in the form of the CC motif (Figure 4c), raises the possibility that specific motifs facilitate the loading of a processive DNA methyltransferase onto DNA in *O. trifallax*. The fact that methylation of all cytosines can extend for hundreds of base pairs (1 kb, for TBE1 transposons, was the maximum in our dataset; Additional file 4e) suggests that the enzyme may stably associate with DNA. It is still a mystery how these methylated MIC sequences are targeted, since they do not contain the CC motif identified in the meDIP-seq analysis. Because MIC-limited repetitive sequences are difficult to assemble, we cannot exclude the possibility that the CC motif or other methyltransferase-recruiting motifs might be present in the nearby genomic context, or that there might be some other targeting mechanism for these sequences.

The use of cytosine-retaining oligos for bisulfite-PCR raised a concern that non-converted unmethylated DNA present at a low level in bisulfite-treated samples might have been erroneously identified as methylated. We addressed this concern in two ways: first, we always performed bisulfite-PCR on vegetative DNA as control (from strain JRB310, JRB510 or both; Figure 6a,b), and second, we used qPCR to quantify the level of signal in a given bisulfite-treated sample, which was compared to a known unmethylated control. Our results were consistent: vegetative DNA never amplified detectable levels of product in ten different primer sets (Figure 6a,b). Similarly, qPCR of bisulfite-treated vegetative DNA generally gave threshold cycle (Ct) values close to a water control (35 to 38 cycles) and close to a non-methylated PCR product used as a bisulfite conversion control (Figure 7a). The 2^-ddCt method for measuring differential qPCR signal is ideally suited to quantitative measurement of both methylated 46 h DNA and unmodified residual DNA from vegetative *O. trifallax *samples [63]. As a proof-of-concept for the use of bisulfite-qPCR for *O. trifallax*, we normalized each bisulfite-treated qPCR cycle count value to itself in native, non-bisulfite converted form.

The 2^-ddCt method quantifies the loss of qPCR signal induced in a sample by bisulfite treatment, and the difference in this loss between vegetative and 46 h DNA constituted the methylation signal in the sample. It is clear from the data plotted in Figure 7a that many samples yielded signals between 30- and 1000-fold higher in 46 h DNA than in vegetative DNA. To consider a chromosome validated, we required a statistically significant difference (*p *< 0.05, Student's one-sided *t*-test for unequal variance) in signal between 46 h and vegetative control (Figure 7a; boldface in Additional file 1). In total, these methods validated 5 of 11 methylation cohort chromosomes at a statistically significant level (including Contig5413.0, which initially failed to amplify by ordinary bisulfite-PCR (Additional file 1)).

In addition to the validated methylation cohort chromosomes, bisulfite-qPCR confirmed methylation in two additional MAC chromosomes and two MIC loci: telomere-end binding protein *α *(*TEBPα*, *p *< 0.05) and *TEBPβ *from the MAC, and TBE1 (*p *< 0.05), and the 170 bp satellite repeat (*p *< 0.05) from the MIC (Figure 7a). The mitochondrial rRNA locus was neither methylated nor hydroxymethylated, and therefore it served as a load control in all subsequent analysis. We attribute the noise observed in the methylation cohort member Contig604.1 to non-specificity of the primers, although we note that the overall trend suggested methylation. Contig4510.0 was a negative control, predicted to be neither methylated nor hydroxymethylated, based on the meDIP-seq data, but its primers appear nonspecific, making the qPCR data difficult to interpret.

*TEBPα *was not expected to show cytosine methylation, since it was not included in any meDIP-seq cohort. Further analysis (described in the next section) revealed that this methylation appears most likely to be specific to aberrantly processed isoforms. In contrast to *TEBPα*, *TEBPβ *was predicted to be hydroxymethylated in the meDIP-seq experiment, and this was validated by bisulfite-qPCR, with approximately 50-fold more signal in 46 h DNA (Figure 7a). All primer sets used in qPCR were designed to cover peaks observed in the meDIP-seq data (Figure 5a, qPCR primers shown as grey arrows), demonstrating that these data are a rich resource for investigating DNA methylation. For example, Contig5413.0 demonstrated no signal in standard bisulfite-PCR (Figure 6b; using the teal-colored primers marked in Figure 5a); however the more closely spaced qPCR primers shown in grey in Figure 5a did detect evidence of methylation (Figure 7a), suggesting that the DNA modifications may be quite localized (within a few hundred base-pairs). Similarly, bisulfite-qPCR of *TEBPβ *only recovered a product when primers targeted the meDIP-seq peaks corresponding to predicted modifications (Figure 5c, grey arrows, and Figure 7a). We attribute the success of these bisulfite-qPCR assays to both the sensitivity of qPCR and the relocation of primers to specific sites predicted to be modified in the genome.

### DNA methylation or hydroxymethylation marks aberrantly rearranged molecules

Despite the fact that *O. trifallax *genome rearrangements are exquisitely programmed by noncoding template RNAs[64], errors of rearrangement occur with surprising abundance during early macronuclear development (but not in mature cells) [64,65], and so produce an additional class of DNA that requires elimination. Typical aberrant rearrangement products include internal deletions within a macronuclear chromosome, when regions that are normally retained are instead effectively treated as if they were precursor (micronuclear-limited) sequence, and removed as a false IES [64,65]. Both programmed and aberrant DNA rearrangement in *O. trifallax *occur between regions of microhomology known as pointers [64,65], and this leads to deletion of the intervening DNA sequence and retention of one copy of the pointer. Such errors can be deleterious when portions of coding sequence are removed or frameshifts are produced; however, the high DNA copy number in the MAC means that copies of correctly-processed genes may also be present.

Bisulfite-PCR recovered three different aberrant *TEBPα *rearrangement products from 46 h DNA, using primers spanning a shorter, scrambled precursor region for this gene (MDS 12 to 2; Figure 6d). In all three cases, IES6, which is normally removed via unscrambling of this locus, was deleted as a conventional IES instead, with subsequent rejoining of flanking DNA at different 3-4 bp cryptic pointers but no segment reordering (Figure 6d). Importantly, all three of these aberrant products displayed evidence of methylation (Figure 6d). Clone 1 was relatively hypomethylated in the surveyed region, but one G was retained, signifying a methylated cytosine on the reverse strand. Further, the amplification of the product from bisulfite-treated DNA suggests that the primer binding sites were also mostly methylated, although this information is absent from the sequencing data because primer binding site methylation cannot be sequenced from PCR products. We infer that clones 2 and 3 derived from heavily methylated regions, because all cytosine and guanosine residues other than the primer binding sites were present and thus methylated.

Consistent with these observations, bisulfite-PCR from 40 h cells recovered a fourth aberrant product from another gene, *TEBPβ*, that appeared to be methylated or hydroxymethylated at most cytosines. This product also displayed the same features of recombination between cryptic pointers (not shown) that are typical for aberrant rearrangement products; however, its sequence was similar but not identical to another aberrant rearrangement product studied in our laboratory, and so we have weaker confidence in this example.

Bisulfite-PCR of *TEBPα *from MDS14 to 15, on the other hand, produced an unexpected, faint double band from 46 h DNA (Figure 6b). Cloning and sequencing indicated that the double band represented both the MIC (longer) and MAC (shorter) versions of the gene, and that both were highly methylated (or hydroxymethylated) (>70%). However, it is possible that this methylated portion of micronuclear sequence was derived from an aberrantly spliced product rather than the micronucleus itself, since we cannot infer the structure of flanking DNA segments from PCR products. Althrough this example could be a rare case of methylation in the MIC genome during development, we favor the simpler model that the bisulfite-resistant MAC and MIC versions we detected were derived from stalled aberrant rearrangement products that amplified with the MDS 12 - MDS 2 primer pair.

### Treatment with azacytidine and deoxyazacytidine inhibits elimination

Overall, the above findings are significant because they link three types of eliminated DNA (parental macronuclear chromosomes, aberrantly rearranged chromosomes and repetitive germline-limited sequences) to conjugation-specific cytosine methylation/hydroxymethylation, suggesting that a functional role for these DNA modifications might be to mark sequences for elimination. We therefore performed experiments designed to test the functional role of DNA methylation during the genome rearrangement process.

Inhibitors of DNA methylation have been developed and used as therapeutic agents against myelodysplastic syndrome, or preleukemia in common parlance [66-69]. The most well known of these agents are azacitidine and decitabine, a nucleoside analog and a deoxynucleoside analog, respectively, of cytidine. Both drugs function by becoming incorporated into the DNA during replication, but they cannot be methylated because they contain a nitrogen atom at position 5 in the pyrimidine ring, preventing the addition of a methyl group at this site. Importantly, azacitidine and decitabine also form covalent adducts with DNA methyltransferases, which leads to proteasomal degradation of these enzymes and, consequently, results in a general block of the DNA methylation process [70]. It is expected that inhibition of DNA methylation would also lead to reduced DNA hydroxyl-methylation, since the latter forms through oxidation of pre-existing methyl groups, and so is dependent on the presence of methylation [41]. Given our hypothesis that DNA methylation in *O. trifallax *has a function in the genome rearrangement process, we asked whether treatment of cells with drugs to inhibit DNA methylation would, in turn, inhibit DNA elimination.

Because the effects of inhibiting DNA methylation in human tissue culture have been extensively characterized (see, for example, [22,23,71,72]), the drug concentrations needed for biological effects in tissue culture were already available to us. First, we tested whether treatment of *O. trifallax *cells with decitabine (0.1 μM, 0.4 μM, or 1 μM) could induce demethylation of validated methylation cohort chromosomes. Cells were grown vegetatively for 48 h either in untreated media or media suplemented daily with fresh decitabine, which has a relatively short half-life in aqueous solution. As expected, we did not observe any morphological defects during vegetative growth, consistent with a lack of detectable DNA methylation during this portion of the lifecycle (Figures 2a, 6a,b and 7a). Next, we induced conjugation by mixing mating types, and at this point we added fresh decitabine to the cultures. Cells were allowed to progress through genome rearrangements (no additional drug was added during the conjugation and rearrangement process) and assayed at 40 h for methylation status. Because our data suggest that methylation levels vary with developmental stage (Figures 2a,b, 3b,c, 6a,b and 7a), it was important to measure the stages of these cells precisely. The staging of the cells was quite consistent between treatments (Figure 7b) with approximately 60% S3 and approximately 30% S4 cells in each sample. We conclude that these concentrations of decitabine do not induce developmental delay or arrest.

To assess methylation in the decitabine-treated cells, DNA was extracted from the 40 h populations shown in Figure 7b, bisulfite treated and assayed by qPCR of various methylated/hydroxymethylated loci. Of 11 tested loci, 7 displayed a statistically significant reduction of DNA methylation in at least one decitabine-treated sample (Figure 7c); one of these loci was the 170 bp satellite repeat shown to be hydroxymethylated by both meDIP-seq and bisulfite sequencing (Additional file 3, Figure 6a,b). The methylation levels of 4 out of 7 tested methylation cohort chromosomes were significantly reduced. The top candidate, Contig2927.0 (Additional files 1 and 3), only displayed a statistically significant demethylation in the 1 μM-treated sample, and this reduction was relatively mild. However, Contig15988 (Alba protein), Contig18539 (collagen) and *TEBPβ *were more robustly demethylated. Contig10532 (unknown protein) showed a possible trend toward reduced methylation but was not statistically significant in this experiment, and methylation levels of Contig11442.0 (6× RRM-containing) were unaffected by decitabine treatment, although it is possible that higher doses of drug or longer treatment length might have produced an effect. We also observed a modest accumulation (1.5- to 2-fold) at 40 h of the same chromosomes whose methylation was reduced (native DNA in Figure 7c, Additional file 1). Four chromosomes had statistically significant (*p *< 0.05) increases in DNA copy number, while five more showed an increase that was not statistically significant (Figure 7c). Together, these data suggested a possible coupling between DNA methylation and degradation, although at the tested drug concentrations the block of methylation, and hence degradation, was incomplete. Overall, the results were consistent with the fact that the cells did not arrest in development (Figure 7b).

**Figure 8:**
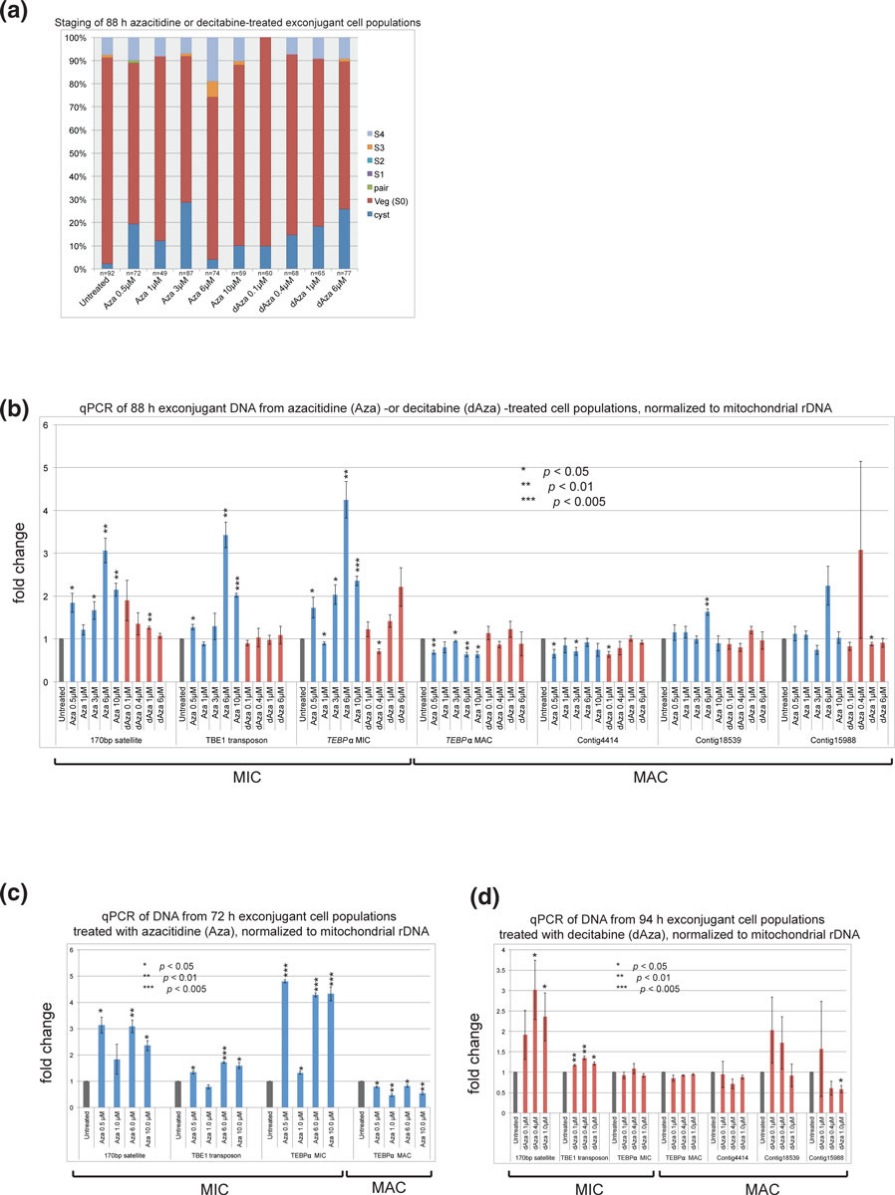
**Effects of decitabine and azacitidine treatment on genome rearrangement in *Oxytricha trifallax***. **(a) **Staging of 88 h exconjugant cell populations treated with azacitidine or decitabine, using criteria from Figure 2e. **(b) **qPCR to measure relative levels of MIC or MAC loci in the same cells staged in (a). Blue bars represent azacitidine-treated populations, red bars represent decitabine-treated populations, and grey bars are untreated controls, set to 1. All qPCR was performed in triplicate and the average is plotted ±standard error. The Student's 1-tailed *t*-test for unequal variance was used to measure statistical significance (*, *p *< 0.05; **, *p *< 0.01, ***, *p *< 0.005). **(c) **Replicate experiment showing reproducible accumulation of MIC DNA, and less processed MAC DNA, in azacitidine-treated exconjugants. **(d) **Replicate experiment showing reproductible accumulation of micronuclear MIC DNA and less MAC DNA in decitabine-treated cell populations. All labeling is as in (a). MAC, macronuclear; MIC, micronuclear; qPCR, quantitative PCR; Veg, vegetative.

We next tested whether inhibition of DNA methylation would cause long-term retention of genomic sequences in exconjugant cells. This time we included azacitidine in the drug regimen along with decitabine, and we allowed the cells to complete genome rearrangements, harvesting them at 88 h post-mixing, when rearrangements are largely complete (Figure 8a). For each population, we assessed the proportion of cells in each stage as a measure of developmental lag (Figure 8a) and observed that vegetative cells or cysts accounted for 90% of most populations, while 10% of cells were still in S4. The 6 μM azacitidine-treated cells did display a slight developmental delay (Figure 8a); however, since no delay was observed in the 10 μM-treated cells, this may not have been related to the drug treatment, or this may have been the optimal concentration for the drug to have effect. Nevertheless, the lagging cells in the 6 μM culture might have increased the germline sequence abundance and so this sample must be interpreted carefully. The other samples did not contain developmentally delayed cells (Figure 8a).

We observed a strong azacitidine-induced retention of the *TEBP∞ *MIC gene and the two repetitive MIC-limited elements (170 bp satellite and TBE1 transposon) (Figure 8b). While some of this effect might have been due to delayed development in the 6 μM sample, delay cannot account for the observed accumulation in other samples (Figure 8b). Furthermore, the 6 μM azacitidine sample also displayed a surprising accumulation of two MAC chromosomes (Contig 18539 and Contig15988), which is unlikely to result from developmental delay. These may instead represent a retention of parental MAC chromosomes that were not eliminated, due to methylation defects in caused by azacitidine treatment.

Decitabine showed a weaker effect on repetitive DNA (Figure 8b). There was a mild retention of the 170 bp repeat but no effect on the TBE1 transposon; we also observed a retentive effect at the *TEBPα *MIC locus. We have performed this experiment twice and observed consistent retention of the 170 bp satellite repeat (Figure 8d; these data are from the experiment in Figure 7c that confirmed demethylation). However, TBE1 retention with decitabine treatment was not consistent (compare Figure 8d to 8b). Retention of the 170 bp satellite was observed independently three times in exconjugant cells treated with azacitidine (two of these experiments are shown in Figure 8b,c and Additional file 5).

Several non-repetitive sequences showed consistent accumulation with drug treatment. The scrambled MIC version of *TEBPα *showed consistent elimination defects in all three experimental replicates of azacitidine treatment (2 of 3 replicates are shown in Figure 8b and 8c), although this effect was not observed in either decitabine experiment (compare Figure 8b,d to Figure 8b,c). Azacitidine induced an accumulation of Contig15988 in both experiments in which it was surveyed (only one shown, Figure 8b), while treatment with decitabine resulted in decreased levels of the same chromosome (Figure 8d).

Some MAC chromosomes consistently exhibited a mild decrease in DNA copy number in exconjugants. The MAC version of *TEBPα *was depleted upon azacitidine treatment in three independent experiments, and a decrease in Contig4414.0 was observed in two azacitidine experiments (it was not measured in the third experiment) (Figure 8b and not shown). We interpret these results as evidence of partial stalling during genome rearrangement, with a failure to regenerate the correct levels of several new macronuclear chromosomes.

## Discussion

DNA methylation plays pivotal roles in development and cell lineage differentiation in plants and animals [1,3-6]. While our knowledge of DNA methylation pathways in animals, plants and fungi is relatively advanced, very little is known about DNA methylation in microbial eukar-yotes, such as ciliates. Though early work uniformly failed to identify cytosine methylation in *Paramecium aurelia, T. thermophila*, or *O. trifallax *[73-75], we have here identified both methylcytosine and hydroxymethyl-cytosine as vital players in the genome rearrangement process of *O. trifallax*. We have unambiguously identified these modifications using high-sensitivity nano-flow UPLC-MS, and have tested their functionality by preventing their formation using methyltransferase inhibitors. Because earlier work examined vegetative samples of *O. trifallax*, which we confirm are lacking in both methylcytosine and hydroxymethylcytosine, it did not detect the *de novo *methylation and hydroxymethylation that we show occurs only transiently during genome rearrangements. Supporting these observations, a report in 2003 described *de novo *methylation in the stichotrichous ciliate (and close *O. trifallax *relative) *Stylonychia lemnae *[76]. In that work, though detected at low levels in vegetative MIC, cytosine methylation was detectable primarily during the genome rearrangement processes, where it was introduced *de novo *within eliminated transposon-like sequences [76]. As in the *O. trifallax *system, methylation was observed in all sequence contexts within the transposable element, and was clustered in a region spanning approximately 500 bp [76]. While our results generally support the conclusions of the *S. lemnae *study, our work differs in some important ways: firstly, since hydroxymethylation had not yet been identified as an important epigenetic mark in DNA, it was not analyzed in *S. lemnae*; secondly, *O. trifallax *DNA methylation/hydroxymethylation occurs at a much higher level (70%-90%) than reported in *S. lemnae *(25%); thirdly, *O. trifallax *has significant modification of at least a few macronuclear chromosomes and aberrant splicing products, neither of which was reported for *S. lemnae*; fourthly, the data presented here directly implicate methylation/hydroxymethylation in *O. trifallax*'s DNA elimination pathway; and, fifthly, we report a 20 bp motif that appears to play a role in directing methylation/hydroxymethylation to particular regions of specific chromosomes. We demonstrate that the DNA methylation process plays a significant functional role in the elimination of repetitive sequences in the MIC, including a highly abundant transposon family and an abundant satellite repeat family. We also report the specific methylation/hydroxymethylation of a small number of aberrantly rearranged molecules but not their correctly rearranged counterparts, suggesting a role for DNA modification in either error recognition during chromosome rearrangement and/or the degradation of such incorrectly rearranged molecules.

Functional data presented here support a role for DNA methylation in degradation pathways, because methylation appears enriched in DNA from the parental MAC, which is targeted for elimination, as well as repetitive MIC eliminated sequences. We found that inhibition of DNA methyltransferases by decitabine led to significant demethylation of 6 out of 9 MAC chromosomes and one MIC locus (the 170 bp satellite repeat; Figure 7c). Coincident with the decitabine-induced loss of methylation from these chromosomes, we observed a mild but often statistically significant accumulation of the chromosomes themselves (the native DNA signal in Figure 7c). While this accumulation is modest, with a maximum 1.5- to 2-fold increase, these data provide compelling support across multiple chromosomes for an intimate link between DNA methylation/hydroxymethylation and degradation during genome rearrangement.

Further support for the model comes from the examination of cells that have completed genome rearrangements after azacitidine and decitabine treatment: 170 bp satellite repeats and TBE1 transposons display a statistically significant accumulation relative to untreated controls (Figure 8b,c,d). In addition, azacitidine treatment induces an accumulation of germline *TEBPα *and a decrease in MAC versions of the same gene (Figure 8b, c). We observe other genome rearrangement defects upon azacididine or decitabine treatment: along with *TEBPα*, Contig4414 also shows lower levels, while two other chromosomes showed elevated levels (Contig18539 and Contig15988), consistent with retention of parental MAC chromosomes that were not degraded properly. These data demonstrate the complexity of the functional consequences of inhibiting DNA methylation: effects may be direct (such as a failure to degrade a given molecule of DNA from the parental MAC) or indirect (for example, if the cell cannot properly eliminate an IES from the MIC version of a gene and therefore does not produce enough MAC product). Further work is needed to disentangle these effects but, taken together, the data implicate a DNA methylation/hydroxymethylation pathway in the elimination of repetitive and single-copy elements from the MIC genome and in the production of a functional macronuclear genome.

The relationship between cytosine methylation and hydroxymethylation in *O. trifallax *offers new challenges. In mouse, for example, sperm DNA is methylated but paternal genome methylation is rapidly lost upon fertilization [77], as the embryo undergoes epigenetic repro-gramming and establishment of new methylation patterns [78,79]. Hydroxymethylcytosine appears in the paternal, but not maternal, pronucleus during this dramatic re-writing of the epigenetic code [80,81], coincident with the loss of paternal methylation. Other work has linked hydroxymethylation with tissue-specific promoter activation and, presumably, demethylation during development [82]. Hydroxymethylation is dependent upon pre-existing methylation and so exists in a dynamic tension with it: both modifications can mark the same genomic regions [83], as we see in *O. trifallax*, and this phenomenon is particularly prevalent in embryonic stem cells [84,85]. Yet hydroxymethylation also antagonizes methylation by directing its removal and/or blocking methylcytosine-binding heterochromatin proteins [86,87]. The link between methylation and degradation in *O. trifallax *suggests that the organism might use hydroxymethylation as a countervailing, stabilizing force, perhaps to target genes that are important for conjugation. Other mechanisms may also be involved in this association: *O. trifallax*'s most hydroxymethylated ribosomal protein gene is a homolog of L12, which in bacteria and yeast can regulate ribosome initiation and elongation [88,89]. Therefore, changes in expression of the L12-encoding chromosome may have ramifications across the proteome, possibly even shutting down translation while the organism undergoes the elaborate steps of genome remodeling.

## Conclusions

In conclusion, we have observed three different types of DNA marked with cytosine methylation in *O. trifallax*: chromosomes targeted for degradation in the parental macronucleus, micronucleus-limited repetitive elements and aberrantly spliced gene rearrangement products. Azacitidine or decitabine treatment significantly inhibited the elimination of at least some sequences, coincident with reduced methylation. The work presented here reveals a novel pattern of extensive cytosine DNA methylation and suggests a functional link to DNA degradation, while also providing a first glimpse into a methylation-based error-detection pathway in *O. trifallax*.

Previous literature reporting the absence of methylctyosine in ciliates [73-75,90] makes our results surprising. However, these earlier studies exclusively surveyed vegetative cell DNA, whereas a more recent study that examined conjugating DNA [76] did detect low levels of methylcytosine, although (as discussed above) no functional role was defined and the levels of modification were much lower than reported here. We do not detect any homologs of the canonical DNA methyltransferase genes (DNMT1, 3A, 3B, or 3L, Dim-2, or the plant-specific methyltransferases CMT3, DRM1/2, and MET1) [1,3,4,91] in the macronuclear genome of *O. trifallax *[92], suggesting that the enzyme might be encoded in the micronucleus or on a rare macronuclear chromosome that was excluded from the macronuclear genome assembly (Swart *et al*., manuscript in revision). However, a search of the current draft micronuclear genome which contains 95% of macronuclear sequences (J.R.B, L.F.L. and X. Chen, unpublished data) revealed no additional candidate methyltransferases.

In contrast to the absence of DNA methyltransferases, we can identify a macronuclear family of Tet (Ten-eleven translocation) dioxygenases resembling those involved in hydroxymethylation in other systems [41,85], as well as a homolog of the DNA methyltransferase-binding protein (DMAP1) [93]. This protein has been reported to activate DNMT1 at sites of DNA breakage during homologous recombination [94]. The association of DMAP1 with an unknown DNA methylase could help *O. trifallax *distinguish between aberrantly spliced products and their correct versions, ensuring that only the former are methylated.

The observation that DNA rearrangements in *O. trifallax *and *S. lemnae *are rife with errors during early nuclear development [65] necessitated a mechanism to correct or eliminate aberrant products, and prompted our laboratory's previous discovery of the long, noncoding RNAs that supply templates for error correction [64]. While the mechanism by which the cell detects aberrantly spliced DNA is unknown, DNA methylation, perhaps coupled with non-coding RNA guides, provides an elegant mechanism to mark DNA splicing mistakes for future degradation, along with other genomic sequences to be eliminated.

## Materials and methods

### *O. trifallax *culture

*O. trifallax *mating types JRB510 and JRB310 were cultured separately in Pringsheim media, the volume of which was doubled every day, and with *Chlamydomonas reinhardtii *as a food source, supplemented daily with fresh overnight cultures of *Klebsiella pneumoniae*. For conjugations, very lightly starved cells were gauze filtered to remove algae clumps. A very small amount of *K. pneumoniae *culture was added (approximately 5 μl in a 300 ml dish) to promote conjugation. The cells form maximal pairs at approximately 12 h post-mixing and separate by 24 h post-mixing. The cells were harvested by killing with 25 mM EDTA, centrifuged for five minutes at 5,000 rpm, and resuspended in buffer T1 of the Nucleospin Tissue Kit (#740952.250, Macherey-Nagel, Bethlehem, PA, USA). The standard protocol was followed for DNA preparation (proteinase K treatement, lysis and purification over a column).

### Immunofluorescence

Twelve-well slides were incubated overnight with 20 mg/ml polylysine in a moist chamber. The wells were washed with water and fixed *O. trifallax *cells (4% paraformaldehyde, 10 to 15 minites, 2× PBS wash) were allowed to adhere to the slides overnight. Cells were permeabilized for 20 minutes with 0.5% Triton-X100 in PBS, then incubated for five minutes with 0.1 N HCl. After washing again, the Image-iT signal enhancer (cat#I36933, Invitrogen, Grand Island, NY, USA) was added to the cells for 30 minutes. The cells were washed, incubated with primary antibody for 1 h at room temperature, then washed for 20 minutes, followed by secondary antibody incubation (1:800 goat anti-mouse or -rabbit; labeled with AlexaFluor 488 or 568) for 1.5 h at 37°C. The cells were washed twice more with PBS, then stained with DAPI (1 to 2 ng/μl in PBS) for two to three minutes. Cells were washed three times with PBS, then mounted with Aqua-Poly/Mount (Polysciences, Warring-ton, PA, USA), and a coverslip was added. Imaging was performed with a confocal microscope at the Princeton University Microscopy Facility. A mouse anti-methylcyto-sine antibody (33D3, ab10805, Abcam, Cambridge, MA, USA) was used at 1:100 dilution and a rabbit anti-hydro-xymethylcytosine antibody (#39792, ActiveMotif, Carlsbad, CA, USA) was used at 1:500 dilution.

### Methylcytosine immunoprecipitation and deep sequencing

All steps were performed as described [44]. We used a Covaris machine (Covaris, Woburn, MA, USA) for fragmentation of 10 μg of 46 h or an equal mixture of JRB310 + JRB510 DNA to 300 bp (10% duty cycle, intensity = 4,200 cycles per burst, 80 seconds). We performed Illumina library adaptor ligation (Illumina, San Diego, CA, USA) before immunoprecipitation, using different indexes for 46 h and vegetative DNA; these libraries were mixed prior to immunoprecipitation and no PCR steps were performed until after IP to avoid loss of DNA modifications. The pooled libraries were subjected to immunoprecipitation using the same anti-methylcytosine and anti-hydroxymethylcytosine antibodies used for immuno-fluorescence, described in the previous section, or control IgG (mouse). Immunoprecipitation was carried out with Dynabeads Protein A (Invitrogen, Grand Island, NY, USA) and a magnetic capture system. After immunoprecipitation as in [44], PCR amplification of the captured material was performed with Illumina adaptor primers by Phusion Hot Start Flex (New England Biolabs, Ipswich, MA, USA) (15 cycles, 60° annealing, 30 second extend), followed by size-selection on a 2% MetaPhor (Cambrex, East Rutherford, NJ, USA) agarose gel to eliminate adaptor dimers and size-select the library. Sequencing was performed on a HiSeq2000 (Illumina, San Diego, CA, USA) at the Princeton University Microarray Facility and approximately 5 to 8 million sequences were obtained for each library. Data were mapped with BWA [95] and the resulting SAM files were custom parsed with custom Python scripts to produce the scatter plots shown in Figure 4. Both raw read files and processed data files are available on Gene Expression Omnibus (GEO); see Data Availability section for more information.

### Treatment of *O. trifallax *with azacitidine and decitabine

Cells were grown and cultured as described above, with separate dishes for each drug concentration. Every day a fresh 500 μM stock of azacitidine or decitabine was prepared by mixing 0.001 g of powder into 10 ml of Pringsheim media, and shaking gently for two to five minutes. This stock was diluted into the culture dishes directly when the cells were fed daily or when conjugation was initiated. Cells were fed the equivalent of 25 ml *C. reinhardtii *culture per 100 ml petri dish (containing 25 ml dense ciliate culture), washed twice in Pringsheim media, and supplemented with 30 μl of fresh *K. pneumoniae *culture. Each day the volume was doubled with fresh Pringsheim media, and the amount of food was doubled. On the third day, the cells were filtered through gauze to remove algae clumps and supplemented with an equal volume of fresh Pringsheim and 5 μl *K. pneumoniae*. Conjugation efficiency and synchrony were quantified by fixing and DAPI staining some cells for microscopy: approximately 700 μl of cells were mixed with an equal volume of a 3:1 Methanol: Acetic acid solution, and allowed to fix for at least five minutes. The cells were gently spun down at 200 *g *for one minute, following which the supernatant was discarded. A volume of cells between 20 and 50 μl was pipetted onto labeled glass coverslips and allowed to air-dry completely (approximately 45 minutes). Once dry, they were either stored or immediately rehydrated and imaged. The rehydration was carried out by floating the coverslips, inverted, for three minutes on the surface of TE buffer, pH 7.5 (10 mM Tris, 1 mM EDTA) in a petri dish. After three minutes the coverslips were removed and excess fluid blotted off, and 20 μl of a 5 μg/ml DAPI solution was immediately applied. The coverslips were inverted onto a glass slide and the edges sealed with clear nail polish to prevent evaporation, and were imaged immediately at 400× magnification on an inverted microscope. Cells were harvested at 40 h or at 72-94 h with the Nucleospin Tissue kit and the DNA analyzed by PCR or qPCR, as described above.

### Bisulfite-PCR

We used the Cells-to-CpG kit (Invitrogen, Grand Island, NY, USA) and followed the standard protocol provided in the kit. In particular, we used a 3× conversion cycle proto-col (denature, then 65°C for 30 minutes, 95° 1.5 minutes, 65° for 30 minutes, 95° 1.5 minutes, 65° for 30 minutes, then desalt and desulfonate). For qPCR, we ran the samples for 45 cycles on an ABI384 (Applied Biosystems, Carlsbad, CA, USA) under standard cycling conditions. We normalized all samples to mitchondrial rDNA and used the ddCt method [63]. Bisulfite-PCR was carried out with primers listed in Additional file 6 and FastStart Taq (Roche, Indianapolis, IN, USA) using the following program: 1) 95° 30 seconds 2) 95° 30 seconds 3) 55° anneal, 4) 72° extend for 30 seconds, for 35 cycles 5) 72° for 5 minutes, 6) 4° hold. A selection of PCR products were Sanger sequenced by GENEWIZ (South Plainfield, NJ, USA) and the traces are presented as supplemental online material (see Data Availability section).

### Southern hybridization

Exconjugant DNA was separated on an ethidium-bromide-stained 0.3% SeaKemGold agarose gel (Lonza, Walkersville, MD, USA). DNA was depurinated in gel (0.25% HCl, 20 minutes; soaked twice in 0.4 M NaOH for 10 minutes to neutralize) and transferred to Hybond XL membrane (GE Healthcare Life Sciences, Pittsburgh, PA, USA) in 0.4 M NaOH using a Nytran TurboBlotter (Schleicher & Schuell, Keene, NH, USA). Labeled probe was generated via random priming (Prime-It, Stratagene, Santa Clara, CA, USA) of gel purified PCR product from *O. trifallax *strain JRB310. After overnight hybridization at 60°C in Church buffer (0.5 M NaPO4, pH 7.2, 1% BSA, 1 mM EDTA, 7% SDS) the membrane was washed twice in 0.2 × SSC with 0.1% SDS (30 min, 60°C), wrapped in saran wrap and exposed to a phosphorimager screen for 6 h or overnight.

### High-resolution nano-flow UPLC-mass spectrometry

Purified *O. trifallax *DNA was subjected to degradation into nucleosides by treatment with DNA Degradase Plus (Zymo Research, Irvine, CA, USA). Methylcytosine and hydroxymethylcytosine controls were obtained from Zymo Research where they were generated by PCR with the appropriately modified nucleotides. Concentrated digest solutions were diluted to approximately 5 ng/ul using 3% acetonitrile (ACN)/0.1% formic acid (FA) and placed directly into autosampler vials. Samples were subjected to reversed-phase nano-LC-MS and MS/MS performed on a nano-flow capillary ultra-high pressure HPLC system (Nano Ultra 2D Plus, Eksigent, Dublin, CA, USA) coupled to an LTQ-Orbitrap XL hybrid mass spectrometer (ThermoFisher Scientific, San Jose, CA, USA) outfitted with a Triversa NanoMate ion source robot (Advion, Ithaca, NY, USA). Chromatography was achieved using a 75 m × 25 cm column packed in house into a fritted capillary (Integrafrit, New Objective, Woburn, MA, USA) using 1.7 um C18 BEH resin (Waters, Milford, MA, USA). Samples were loaded directly on column and separations were conducted using a linear gradient of A and B solvents (Solvent A: 3% ACN/0.2% FA/0.1% acetic acid; Solvent B: 97% ACN/0.2% FA/0.1% acetic acid) over 60 or 90 minutes at a flow rate of approximately 250 nl per minute. Nano electrospray ionization was carried out using the NanoMate ion source at 1.74 kV, with the LTQ heated capillary set to 200°C. Full-scan mass spectra were acquired in the Orbi-trap in positive-ion mode over the m/z range of 225 to 1800 and the narrower range of 225 to 300 at an instrument resolution of 100,000. Spectra were typically within a calibration error of 2 to 3 ppm with respect to absolute mass accuracy, following external calibration of the instrument. LC-MS data were manually interpreted using Xcalibur software (ThermoFisher Scientific, San Jose, CA, USA) to visualize nucleoside mass spectra and to generate extracted ion chromatograms by using the theoretical [M+H] values of the target nucleosides within a range of ±0.0005 Da. Data are available on OxyDB, the *O. trifallax *genome database website. See Data Availability section for details.

### Data availability

The DNA immunoprecipitation and high-throughput sequencing data are available on Gene Expression Omnibus (GEO) [96]: Accession number [GSE41060]. Sanger traces from bisulfite-PCR sequencing are available as Additional files 7, 8, 9, 10, 11, 12, 13, 14, 15. UPLC-mass spectrometry data are available at OxyDB, the *O. trifallax *genome database website [43].

## Abbreviations

ACN: acetonitrile; bp: base pair; Ct: threshold cycle; DAPI: 4',6-diamidino-2-phenylindole; ddCt: delta-delta-Ct; DMAP1: DNA methyltransferase binding protein; Dnmt: DNA methyltransferase; FA: formic acid; kb: kilobases; MAC: macronucleus; MIC: micronucleus; IES: Internal Eliminated Sequence; IgG: immunoglobulin G; LC: liquid chromatography; MDS: Macronuclear Destined Sequence; MS: mass spectrometry; PCR: polymerase chain reaction; qPCR: quantitative PCR; TBE: Telomere-Bearing Element; TEBPα: Telomere End-Binding Protein α; TEBPβ: Telomere End-Binding Protein β; UPLC: ultra high performance liquid chromatography.

## Supplementary Material

Additional file 1 **The methyl cohort**. A list of chromosomes whose methylation was predicted by meDIP-seq and validated by bisulfite-PCR or qPCR. Columns labeled as 'signal' indicate the number of meDIP-seq reads at 46 h less the number of reads from vegetative DNA. For columns labeled 'bisulfite-qPCR' and '40 h dAza demethylated?', bold font denotes statistically significant effects relative to vegetative or untreated controls (Student's 1-tailed *t*-test for unequal variance, *p *< 0.05), while a 'yes' indicates an effect that was observed but did not reach statistical significance. 'n/a' indicates that the test was not performed. Click here for file

Additional file 2 **The CC motif cohort**. A list of chromosomes containing three or more CC motifs and that overlap substantially with the methylation and hydroxymethylation cohorts (Additional files 1 and 3). Click here for file

Additional file 3 **The hydroxymethyl cohort**. A list of chromosomes whose hydroxymethylation was predicted by meDIP-seq, with signal defined as in Additional file 1. Click here for file

Additional file 4 **Supplemental figure depicting bisulfite-PCR sequence analysis. (a) **Contig4414.0 following bisulfite treatment of 40 h DNA and PCR with C-to-T converted primers, which are not methyl-specific. **(b) **Contig4414.0 following bisulfite treatment of 40 h DNA and PCR with cytosine-retaining (methyl-specific) primers. **(c) **The 170 bp satellite following bisulfite treatment of vegetative and PCR with C-to-T converted primers, which are not methyl-specific. **(d) **The 170 bp satellite following bisulfite treatment of 40 h DNA and PCR with cytosine-retaining (methyl-specific) primers. **(e) **The transposon *TBE1 *following bisulfite treatment of 40 h DNA and PCR with cytosine-retaining (methyl-specific) primers. *TBE1, telomere bearing element 1*. Click here for file

Additional file 5 **Supplemental figure depicting Southern analysis for retention of 170 bp repeat and TBE1 transposon in 72 h azacitidine-treated cells. (a) **Southern hybridization. **(b) **quantification of (a). Click here for file

Additional file 6 **A table of primers used in this study**. All primers used for bisulfite PCR, bisulfite qPCR and Southern blotting experiments. Click here for file

Additional file 7 **Sanger sequencing data (in FASTA format) for Figure **6b. Bisulfite-treated *TEBP**α ***PCR product, MAC isoforms (smaller) shown in lane 9. *TEBP**α***, *Telomere End-Binding Protein **α***. MAC, macronucleus. Click here for file

Additional file 8 **Sanger sequencing data (in FASTA format) for Figure **6b. Bisulfite-treated *TEBP**α ***PCR product, MIC isoforms (larger) shown in lane 9. *TEBP**α***, *Telomere End-Binding Protein **α***. MIC, micronucleus. Click here for file

Additional file 9 **Sanger sequencing data (in FASTA format) for Figure **6c. Contig4414 bisulfite sequences. Click here for file

Additional file 10 **Sanger sequencing data (in FASTA format) for Figure **6d. Bisulfite sequences of *TEBP**α ***aberrantly spliced products. *TEBP**α***, *Telomere End-Binding Protein **α***. Click here for file

Additional file 11 **Sanger sequencing data (in FASTA format) for Additional file **4a. Sequences for Contig4414.0 following bisulfite treatment of 40 h DNA and PCR with C-to-T converted primers. Click here for file

Additional file 12 **Sanger sequencing data (in FASTA format) for Additional file **4b. Sequences for Contig4414.0 following bisulfite treatment of 40 h DNA and PCR with cytosine-retaining (methyl-specific) primers. Click here for file

Additional file 13 **Sanger sequencing data (in FASTA format) for Additional file **4c. Sequences for the 170 bp satellite following bisulfite treatment of vegetative and PCR with C-to-T converted primers. Click here for file

Additional file 14 **Sanger sequencing data (in FASTA format) for Additional file **4d. Sequences for the 170 bp satellite following bisulfite treatment of 40 h DNA and PCR with cytosine-retaining (methyl-specific) primers. Click here for file

Additional file 15 **Sanger sequencing data (in FASTA format) for Additional file **4e. Sequences for the transposon *TBE1 *following bisulfite treatment of 40 h DNA and PCR with cytosine-retaining (methyl-specific) primers. *TBE1, telomere bearing element 1*. Click here for file
