# Supplementary material for: Cytosine methylation and hydroxymethylation mark DNA for elimination in Oxytricha trifallax
Source: Genome Biol. 2012 Oct 17;13(10):R99. doi: 10.1186/gb-2012-13-10-r99 (PMC3491425; doi:10.1186/gb-2012-13-10-r99)

A Contig4414.0 40hr bisulfite-treated DNA Nonmethyl-specific PCR (C-to-T converted oligos)

note: reverse strand tested, G convert to A (if unmethylated)

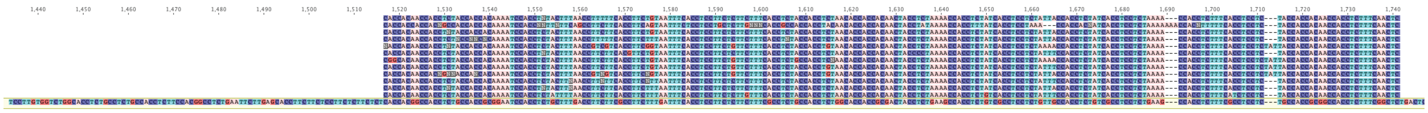

B Contig4414.0 40hr bisulfite-treated DNA Methyl-specific PCR (C-retaining oligos)

Top=forward strand tested, C convert to T (if unmethylated)

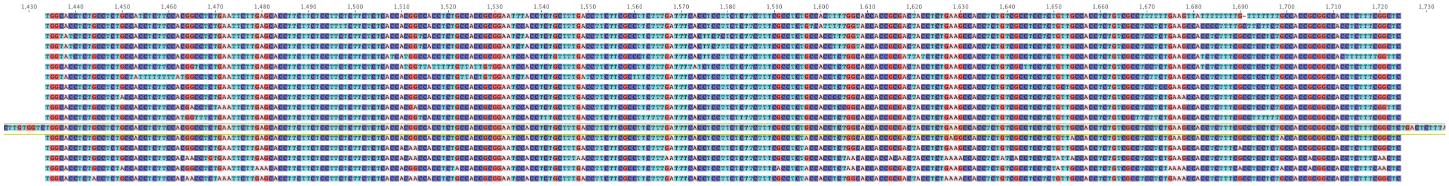

Bottom=reverse strand tested, G convert to A (if unmethylated)

C bisulfite-treated vegetative DNA 170bp satellite non-methyl-specific

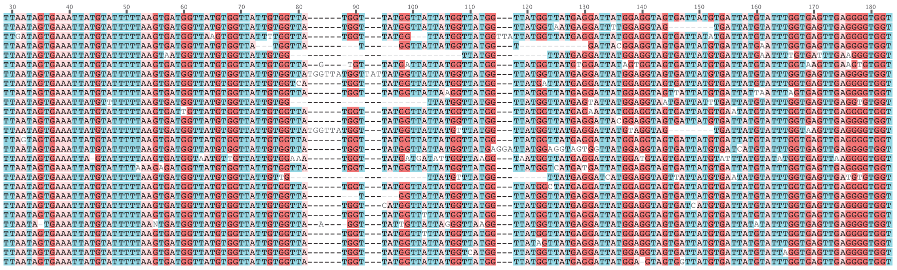

D bisulfite-treated 40hr DNA 170 satellite methylation-specific

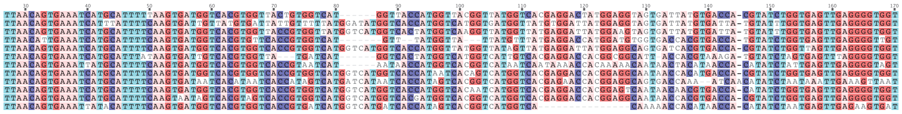

E TBE1 40hr bisulfite-converted DNA, methyl-specific primers

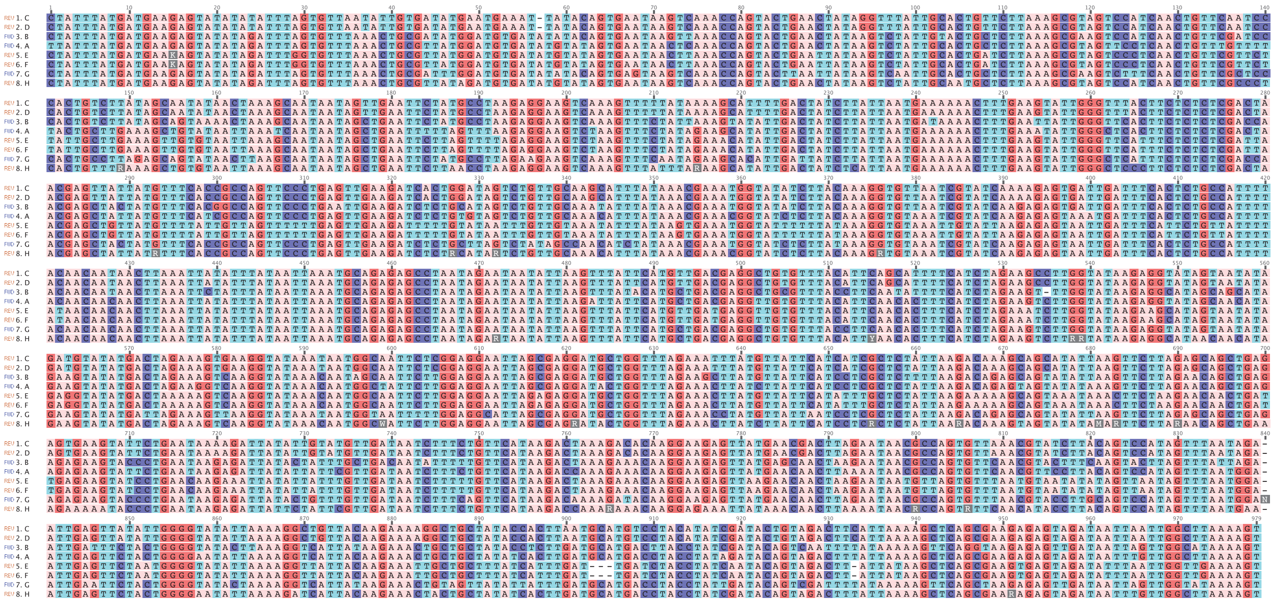

Supplement: Additional file 4 — Supplemental figure depicting bisulfite-PCR sequence analysis. (a) Contig4414.0 following bisulfite treatment of 40 h DNA and PCR with C-to-T converted primers, which are not methyl-specific. (b) Contig4414.0 following bisulfite treatment of 40 h DNA and PCR with cytosine-retaining (methyl-specific) primers. (c) The 170 bp satellite following bisulfite treatment of vegetative and PCR with C-to-T converted primers, which are not methyl-specific. (d) The 170 bp satellite following bisulfite treatment of 40 h DNA and PCR with cytosine-retaining (methyl-specific) primers. (e) The transposon TBE1 following bisulfite treatment of 40 h DNA and PCR with cytosine-retaining (methyl-specific) primers. TBE1, telomere bearing element 1. [file gb-2012-13-10-r99-S4.PDF]
