# Supplementary figures and images for: Cytosine methylation and hydroxymethylation mark DNA for elimination in Oxytricha trifallax
Source: Genome Biol. 2012 Oct 17;13(10):R99. doi: 10.1186/gb-2012-13-10-r99 (PMC3491425; doi:10.1186/gb-2012-13-10-r99)

# A

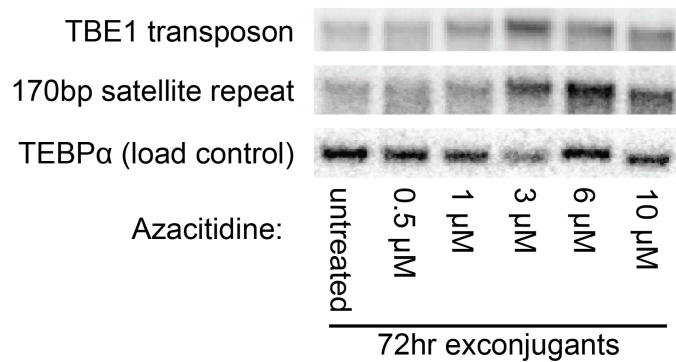

# B

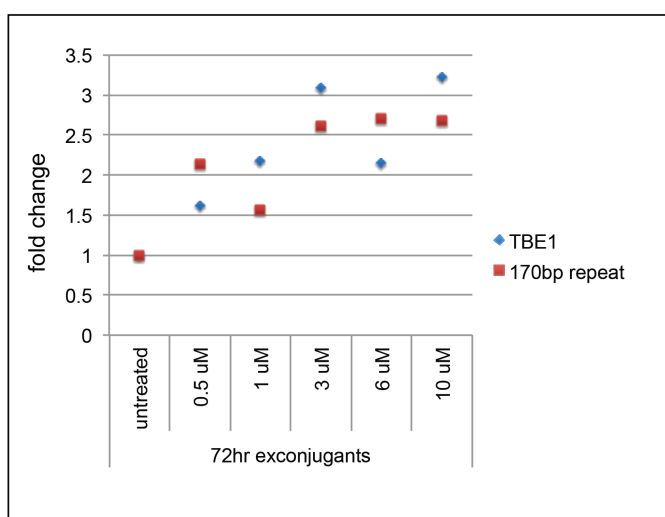

Supplement: Additional file 5 — Supplemental figure depicting Southern analysis for retention of 170 bp repeat and TBE1 transposon in 72 h azacitidine-treated cells. (a) Southern hybridization. (b) quantification of (a). [file gb-2012-13-10-r99-S5.PDF]
